# Assessment of bladder volume by nurses using point-of-care ultrasound: a cross-sectional study

**DOI:** 10.1590/1980-220X-REEUSP-2024-0285en

**Published:** 2025-07-14

**Authors:** Tatiana Fuzaro, Dejanira Aparecida Regagnin, Thales Henrique Vieira Brandão, João Paulo Victorino, Eduarda Ribeiro dos Santos, Filipe Utuari de Andrade Coelho

**Affiliations:** 1 Hospital Israelita Albert Einstein, Departamento de Pacientes Graves, São Paulo, SP, Brazil.; 2 Faculdade Israelita de Ciências da Saúde Albert Einstein, São Paulo, SP, Brazil.; 3 Universidade de São Paulo, Escola de Enfermagem, São Paulo, SP, Brazil.

**Keywords:** Nursing, Ultrasonography, Urinary Retention, Urinary Bladder, Advanced Practice Nursing

## Abstract

**Objectives:**

To identify the main reasons for performing point-of-care bladder ultrasonography (POCUS) by nurses and to compare the volume found on POCUS with the volume of urine drained by a relief bladder catheter (RBC) or delay bladder catheter (DBC).

**Method:**

A cross-sectional study was conducted in intensive care, semi-intensive care, and medical-surgical units with patients over 18 years of age who had no spontaneous urination for more than six hours or for more than four hours after removal of the DBC.

**Results:**

A total of 211 patients were included, with the main reasons for performing POCUS being absence of urination after surgical procedures (35.0%), removal of the DBC (35.1%), decreased level of consciousness (DLC) (13.7%), and DBC obstruction (0.9%). The correlation between POCUS volume and urine volume drained was considered very strong.

**Conclusion:**

Situations such as postoperative, removal of DBC, DLC, and DBC obstruction lead to the performance of POCUS by nurses, with a very strong correlation between the volumes found in POCUS and those drained by RBC or DBC.

## INTRODUCTIOn

Currently, the use of ultrasonography (USG) by nurses, through the concept of point-of-care ultrasonography (POCUS), has gained ground, standing out for its contribution to the management of urinary retention (UR) and the assessment of bladder volume^(1,2,3)^. UR is a complication associated with bladder distension, which can cause damage to the organ, impact the length of hospital stay, and contribute to the development of urinary tract infections (UTIs)^(4)^.

According to the International Continence Society, UR is defined by the presence of a painful, palpable, or percussible bladder when the patient is unable to urinate^(5)^. Volumes greater than 300 mL are often considered the minimum value necessary to identify a distended bladder, except in obese patients^(6)^. The incidence of UR is estimated at up to 6.8 cases per 1,000 person-years in the general male population due to the longer urethra and the presence of the prostate, with an incidence ratio between men and women of 13:1 (6–7). UR is a common cause of morbidity, accounting for more than 30,000 hospital admissions per year in the English healthcare system^(7)^.

The most frequent etiologies of UR include urinary tract obstruction due to intrinsic and extrinsic causes, especially in men, due to benign prostatic hyperplasia (BPH)^(6,7)^. Urinary tract infections (UTIs), commonly caused by Escherichia coli, are also significant^(6,7)^. Neurogenic origin is associated with changes in coordination between the sympathetic and parasympathetic nervous systems, impacting complete bladder emptying^(6,7)^. Finally, iatrogenic UR is related to pharmacological issues involving a variety of medications that alter bladder control.

It is extremely important that UR be identified quickly to avoid complications, which range from psychological distress to hematuria due to rapid decompression and the development of infections. Chronic complications include decreased bladder contractility and sensitivity, incontinence, hydronephrosis, and long-term renal failure^(6,7)^. The most common treatment for UR is bladder catheterization (BC), both for relief (RBC) and delay (DBC). It is important to note that approximately 80% of UTIs in hospitalized patients are related to BC^(6,7,8)^.

In this context, bladder assessment by nurses is of paramount importance to obtain information that can serve as a basis for actions based on the nursing process^(9,10)^. When faced with a nursing diagnosis of impaired urinary elimination, as proposed by NANDA *International*, nurses are responsible for assessing the patient’s clinical data and proposing relevant nursing interventions that promote improvement or stabilization of the situation found^(10,11)^. Recently, the nursing intervention “bladder ultrasound” was developed according to the Nursing Intervention Classification (NIC), which will be included in the next edition of the NIC, as it includes items intended for the practice of POCUS in bladder assessment by nurses^(11)^.

USG is currently the most effective noninvasive method as an auxiliary semiological tool, providing immediate bedside information to confirm physical examination findings, which allows for faster decision making^(12)^. In the case of UR, USG is a reliable method, with 97% sensitivity, 91% specificity, and 94% accuracy^(13)^.

It should be emphasized that the opinion of the Regional Nursing Council of São Paulo (COREN-SP) COREN-SP 029/2014 – CT PRCI No. 1530/2014, together with the Law on the Practice of Nursing (Law No. 7,498, of June 25, 1986), ensures autonomy for the use of USG for the purpose of measuring bladder volume by a trained nurse^(14,15)^. It should also be noted that, in 2021, the Federal Nursing Council (COFEN) issued opinion COFEN No. 679/2021, which regulated the performance of POCUS at the bedside^(16)^. However, the preparation of test reports and nosocomial diagnoses is not the responsibility of nurses, as this is an activity exclusive to medical professionals^(14,16)^.

The use of POCUS by nurses in Brazil is recent and has received attention due to its applicability in various areas of nursing care^(17)^. However, the literature is still scarce regarding USG and nursing practice in measuring bladder volume using POCUS. In a single Brazilian study with adult patients in inpatient units, a strong correlation was found between the volume of urine drained by RBC and the bladder volume found by nurses on USG before RBC^(1)^. Another Brazilian study with critically ill patients showed the usefulness of USG in the detection of UR by nurses, but did not provide detailed information on the practice of bladder POCUS, focusing mainly on factors related to the development of UR^(2)^.

Given the above, we can conclude that the use of USG for measuring bladder volume performed by nurses at the bedside is a quick, non-invasive assessment that allows for more accurate decision-making. Therefore, given the scarcity of national studies that contextualize issues related to the performance of bladder POCUS in nursing practice, further research on this topic is necessary. Thus, the objective of this study is to identify the main reasons for performing bladder POCUS by nurses and to compare the volume identified in POCUS with the volume of urine drained by RBC or DBC.

## METHOD

### Type of Study

This is a retrospective cross-sectional study, prepared following the recommendations of the STROBE checklist to ensure greater transparency and consistency in the presentation of data.

### Location

The study was conducted in a large hospital located in the city of São Paulo, SP, and included three units dedicated to the care of adult patients: the intensive care unit, with 42 general beds; the semi-intensive care unit, with 50 beds; and the medical-surgical units, which have approximately 200 beds.

### Training of Nurses in Bladder Pocus

In 2015, an institutional protocol was developed at the aforementioned institution to measure bladder volume using POCUS performed by nurses. Since then, nurses have been trained to use this tool after being admitted to the institution.

The institutional training of nurses to perform bladder POCUS consists of a two-hour theoretical explanation, which covers essential theoretical aspects for understanding and handling the USG equipment, in addition to the specific content of POCUS. The content also includes a brief anatomical review of the bladder with its anatomical planes for visualization and the technique for measuring bladder volume. It is important to mention that the training group consists of 5 to 10 nurses, with the aim of adequately balancing the number of participants in relation to the number of instructors, as well as the availability of ultrasound equipment and volunteers for practice.

After the theoretical part, there is a structured practical part consisting of hands-on activities, lasting two hours, to develop skills in handling the ultrasound equipment and applying the bladder POCUS technique on volunteers. At this stage, the professionals are accompanied by a nurse who is a POCUS reference at the institution, who also teaches the theoretical part.

During the practical training, two volunteers from the institution itself are used, usually nursing students, who are encouraged to drink water periodically in order to maintain bladder volume for the nurses to perform the exams. It should be noted that professionals are instructed to perform five to ten insonations on volunteers, accompanied by the aforementioned instructor, and after this, they undergo a practical evaluation. This evaluation consists of performing the bladder POCUS technique on one of the volunteers, who may be considered fit or unfit by the instructor, based on a checklist developed by the institution itself that contains the fundamental items of the respective technique. The professional is approved at this stage of the training if they achieve a score of 80% or higher on the checklist. If deemed unfit, the professional is called for further training with future classes and is not allowed to practice at their work unit until they pass. It is important to note that the units chosen for data collection in this study, during the selected period, had nurses with more than six months of experience in bladder POCUS.

According to the institutional protocol for bladder POCUS, in situations where the patient does not have spontaneous diuresis for more than six hours or for more than four hours after removal of the DBC, the nurse must perform a physical examination to assess the presence of a palpable bladder globe, signs of discomfort or abdominal distension, presence of urge to urinate, or agitation and sweating. After this assessment, the nurse should perform bladder POCUS to measure the amount of bladder volume. Depending on the volume found, the institutional protocol suggests emptying maneuvers, such as standing next to the bed, performing the Valsalva maneuver, applying heat to the suprapubic region, promoting the sound of running water, and sitting on the toilet. If these actions are not effective or not indicated by the clinical picture, and the volume obtained in the bladder POCUS is greater than or equal to 400 ml, or above 200 ml accompanied by symptoms of discomfort, the physician is called to discuss the need for RBC. DBC is indicated for volumes greater than 1000 ml found in the bladder POCUS and with previous episodes of UR^(18)^. The definition used in this study to characterize UR was a urine volume above 500 ml found in the bladder POCUS^(2)^.

The ultrasound devices used at the institution are SonoSite M-Turbo® devices, registered with the Brazilian Health Surveillance Agency under No. 80022060089 and licensed for professional use and for the care of the population. These devices are configured to evaluate images using convex, linear, and sector probes. Each unit mentioned above (ICU, semi-intensive care, and medical-surgical clinic) has this equipment available for use by nurses. To perform the bladder volume measurement technique used at the institution, the patient must be positioned in a horizontal supine position. Next, the convex transducer is positioned in the suprapubic region in the transverse plane. At this point, while insulating the bladder, the length of the bladder’s lateral diameter is calculated. Subsequently, the position of the transducer is changed, keeping it in the anatomical location, for visualization in the longitudinal plane. At this stage, bladder insonation should include the calculation of the anteroposterior and craniocaudal diameters. The equipment used at the institution automatically calculates the bladder volume.

### Sample and Selection Criteria

The sample was of convenience, consisting of medical records of patients over 18 years of age, evaluated with bladder POCUS by a nurse, who did not present spontaneous diuresis for more than six hours or after removal of the DBC for more than four hours and had the introduction of RBC or DBC as a post-procedure action, during their hospitalization in the aforementioned units, from January 2017 to December 2018. After identifying the patients through a database, 395 eligible individuals were found. All patients were contacted by telephone and, after presenting the study, the researchers sent the Free and Informed Consent Form (FICF) via the Google Forms platform. Of these, 25 refused to participate and 99 had incomplete records in their medical records, resulting in the inclusion of 211 patients in the study. It should be noted that all eligible patients were contacted.

### Study Variables

The variables collected included demographic information (gender and age), clinical characteristics (diagnosis of hospitalization, medical history, physical examination, and potential medications in use that interfere with bladder emptying) and data related to the performance of POCUS (time without urination, reason for performing USG, urine volume found on USG, procedures after USG, and urine volume drained later by RBC or DBC).

### Data Collection

Data collection was performed by the authors through the review of electronic medical records, after authorization from the participants by signing the FICF. The identification of medical records occurred in January 2020, contact with patients was made between March and April 2020, and data collection through the review of medical records took place between May and June of the same year.

### Data Analysis and Processing

The data were entered into the *Research Electronic Data Capture*® (REDCap®)^(19)^ platform and imported into a *Microsoft Excel* ® 2007 spreadsheet. For the analyses, the *Statistical Package for the Social Science* (SPSS) software, version 26, was used. Descriptive analysis was performed using absolute and relative frequencies for qualitative variables, while for quantitative variables, normality was first tested using the Shapiro-Wilk test. After verifying normality, the mean and standard deviation were calculated. Regarding inferential analysis, the chi-square and Fisher tests were used for qualitative variables and the Student’s t-test for quantitative variables.

To analyze the comparison between the bladder volume observed in POCUS by the nurse and the subsequent urine volume found in RBC or DBC, the *Person* correlation coefficient was used. The level of significance adopted for the analysis was *p* ≤ 0.005.

### Ethical Aspects

This research was in accordance with Resolution No. 466/2012 and was submitted to and approved by the Research Ethics Committee of the aforementioned institution, under approval number 4.533.418 and CAEE: 40058020700000071.

## RESULTS

A total of 211 patients were included, who underwent 211 bladder POCUS by the nurse (one per patient). Table 1 shows the characterization of patients who underwent bladder POCUS by the nurse according to the presence or absence of UR. UR was present in 119 (56.3%) of the patients, more than half of whom were male, with a mean age of 65.1 ± 20.2 years. Systemic arterial hypertension (SAH) was the most common clinical antecedent, and the diagnosis of immediate postoperative hospitalization was more frequent in the group with UR. Among the medications in use with the potential to alter urinary frequency, opioids stood out in patients with UR.

**Table 1:** Characterization of patients who underwent bladder POCUS by nurses according to the presence or absence of UR – São Paulo, SP, Brazil, 2020.

Variable	Total n = 211 (%)	Without UR n = 92 (%)	With UR n = 119 (%)	p-Value
Gender				
Men	123 (58.3)	51 (55.4)	72 (60.5)	0.459^a^
Women	88 (41.7)	41 (44.6)	47 (39.5)	
Clinical background				
SAH	81 (38.4)	41 (44.6)	40 (33.6)	0.105^a^
DM	30 (14.2)	14 (15.2)	16 (13.4)	0.715^a^
COPD	14 (6.6)	9 (9.8)	5 (4.2)	0.106^a^
HF	14 (6.6)	9 (9.8)	5 (4.2)	0.106^a^
CKD	8 (3.8)	4 (4.3)	4 (3.4)	0.710^a^
Diagnosis at admission				
Post operatory	103 (48.8)	35 (38.0)	68 (57.1)	0.006^a^
Neurology	42 (19.9)	24 (26.1)	18 (15.1)	0.048^a^
Respiratory	25 (11.8)	15 (16.3)	10 (8.4)	0.078^a^
Infectious disease	22 (10.4)	9 (9.8)	13 (10.9)	0.788^a^
Cardiovascular	13 (6.2)	4 (4.3)	9 (7.6)	0.335^a^
Urology	12 (5.7)	7 (7.6)	5 (4.2)	0.289^a^
Gastrointestinal	7 (3.3)	3 (3.3)	4 (3.3)	0.748^a^
Pharmacologic group				
Opioids	63 (29.9)	18 (19.6)	45 (37.8)	0.004^a^
Antipsychotics	38 (18.0)	18 (19.6)	20 (16.8)	0.605^a^
Analgesic narcotics	36 (17.1)	16 (17.4)	20 (16.8)	0.911^a^
Antispasmodics	27 (12.8)	14 (15.2)	13 (10.9)	0.355^a^
Antidepressive	14 (6.6)	6 (6.5)	8 (6.7)	0.954^a^
Antiparkinsonians	11 (5.2)	5 (5.4)	6 (5.0)	> 0.999^b^
Death	49 (23.2)	26 (28.3)	23 (19.3)	0.128^a^

UR: urinary retention, SAH: systemic arterial hypertension; DM: diabetes mellitus; COPD: chronic obstructive pulmonary disease; HF: heart failure; CKD: chronic kidney disease, a: chi-square test, b: Fisher’s exact test.

The information related to the performance of bladder POCUS and involved in urination according to the presence or absence of UR is shown in Table 2. The unit that performed bladder POCUS most frequently was the medical-surgical clinic. The most frequent presence of a palpable bladder globe was in the UR group, and the predominant reason for the absence of urination that led to the performance of POCUS by the nurse was associated with surgical procedures. The bladder volume found on POCUS was significantly higher in patients with UR. The main action after performing bladder POCUS was the introduction of a RBC, and the volume of urine drained by the RBC or DBC after POCUS was higher in the UR group.

**Table 2:** Information related to the performance of bladder POCUS and involved in urination according to the presence or absence of UR – São Paulo, SP, Brazil, 2020.

Variable	Total n = 211 (%)	Without UR n = 92 (%)	With UR n = 119 (%)	p-Value
Unit where POCUS was done				
Clinical-Surgical Ward	142 (67.3)	56 (60.9)	86 (72.3)	0.080^a^
Semi-intensive	36 (17.1)	19 (20.7)	17 (14.3)	0.223^a^
ICU	33 (15.6)	17 (18.5)	16 (13.4)	0.318^a^
Time without urinating (hours)				
Mean ± SD	7.5 ± 2.5	7.6 ± 2.4	7.4 ± 2.5	0.586^b^
Palpable vesical globe n (%)	113 (56.3)	38 (41.3)	75 (63.0)	0.002^a^
Causes of absence of urination				
Surgical procedure n (%)	75 (35.5)	23 (25.0)	52 (43.7)	0.005^a^
DBC withdrawal n (%)	74 (35.1)	40 (43.5)	34 (28.6)	0.024^a^
RLC n (%)	29 (13.7)	19 (20.7)	10 (8.4)	0.010^a^
DBC occlusion n (%)	2 (0.9)	1 (1.1)	1 (0.8)	> 0.999^c^
Verifiable volume in POCUS				
Mean ± SD	563.3 ± 251.8	345.0 ± 107.5	732.1 ± 195.0	< 0.001^b^
Action after POCUS				
RBC n (%)	190 (90.0)	81 (88.0)	109 (91.5)	0.544^a^
DBC n (%)	21 (10.0)	10 (10.9)	11 (9.2)	0.696^a^
Volume drained by RBC or DBC after POCUS				
Mean ± SD	593.1 ± 290.3	395.0 ± 167.3	746.3 ± 272.4	< 0.001^b^

UR: urinary retention, POCUS: point-of-care ultrasonography, ICU: intensive care unit, DBC: urinary delay catheter, RNC: reduced level of consciousness, RBC: relief urinary catheter, a: chi-square test, b: *Student’s* T-test, c: *Fisher’s* exact test.

**Figure 1:**
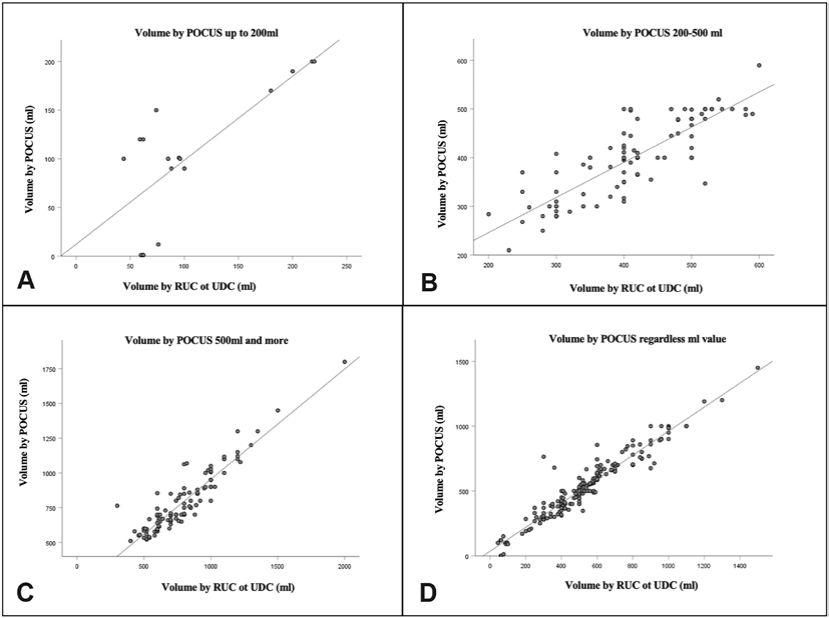
Correlation between POCUS volumes and urine volume verified upon subsequent insertion of the RBC or DBC – São Paulo, SP, Brazil, 2020.

Figure 1 shows the correlation between the volumes estimated by the nurse using POCUS and the urine volume subsequently verified by introducing the RBC or DBC. Figure 1A shows the correlation between the POCUS volume up to 200 ml and the volume measured by the RBC or DBC (r = 0.759, *p* < 0.001). Figure 1B shows the correlation between the POCUS volume from 200 to 500 ml and the volume by RBC or DBC (r = 0.836, *p* < 0.001). Figure 1C shows the correlation between POCUS volume above 500 ml and volume measured by RBC or DBC (r = 0.922, *p* < 0.001). Finally, Figure 1D illustrates the correlation between POCUS volume, regardless of the ml found, and volume by RBC or DBC (r = 0.958, *p* < 0.001).

POCUS: *point of care ultrassonography*, RBC cateter vesical de alívio, DBC: cateter vesical de demora, ml: mililitros.

**Figure 2:**
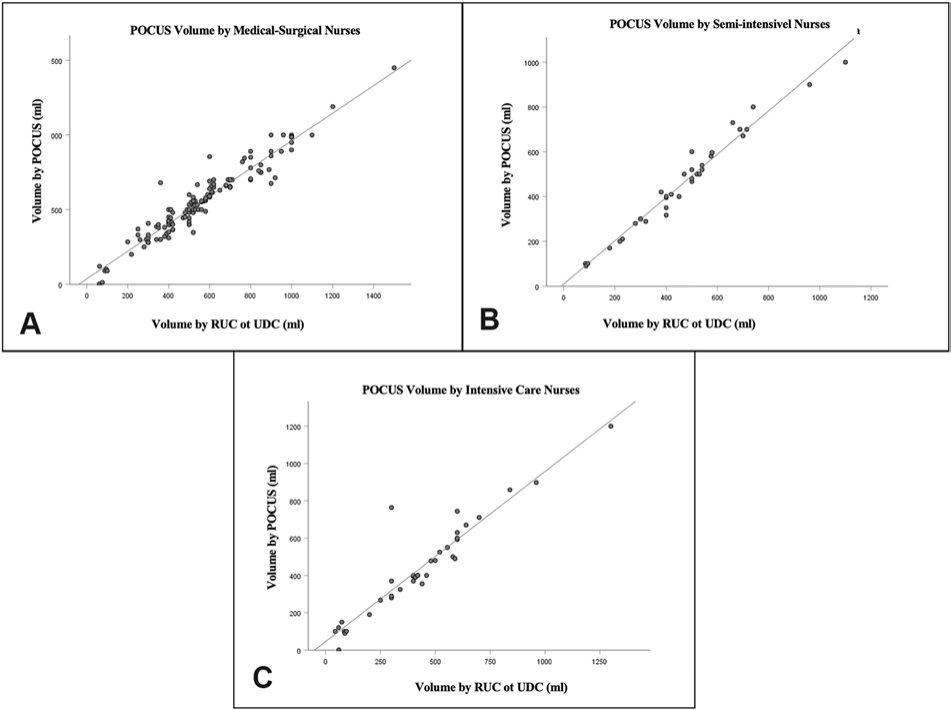
Correlation between POCUS volumes measured by nurses, according to the unit, and urine volume verified upon subsequent introduction of RBC or DBC. Figure 1A shows the correlation between the bladder POCUS volume found by nurses in the medical-surgical clinic, Figure 1B by nurses in the semi-intensive care unit, and Figure 1C by nurses in the ICU – São Paulo, SP, Brazil, 2020.

The correlation between the volumes estimated in the bladder POCUS performed by the nurse, according to the admission unit, and the urine volume verified later through the introduction of the RBC or DBC is shown in Figure 2. Figure 2A shows the correlation between the bladder POCUS volume found by nurses in the medical-surgical clinic and the urine volume drained by RBC or DBC (r = 0.959, *p* < 0.001). Figure 2B shows the correlation between the bladder POCUS volume measured by nurses in the semi-intensive care unit and the volume drained by RBC or DBC (r = 0.985, *p* < 0.001). Figure 2C shows the correlation between the bladder POCUS volume measured by ICU nurses and the volume drained by RBC or DBC (r = 0.940, *p* < 0.001).

POCUS: *point of care ultrasonography*, RBC relief bladder catheter, DBC: Delay bladder catheter, ml: milliliters.

## DISCUSSION

In the present study, it is possible to observe a profile of elderly patients, most of whom underwent some type of surgical procedure requiring evaluation by a nurse because they did not present diuresis, especially after removal of the DBC. The correlation between the volume found in the bladder POCUS by the nurse and the volume of urine drained by RBC or DBC was generally significant, since it changes gradually according to the volume found in the POCUS and does not differ according to the nurse’s workplace.

Regarding the prevalence of UR, more than half of the patients analyzed presented this alteration. A Brazilian review showed that among the main practices used for the diagnosis of UR, the use of USG for decision-making in the face of BC stands out^(20)^. In a Dutch study with patients in the postoperative period of hip and knee arthroplasty surgery, the frequency of UR was significantly lower than in the study^(21)^.

However, a Polish study with patients in the same surgical context found a prevalence similar to that of the study^(22)^. In the national scenario, a study with patients from a medical-surgical clinic presented a lower UR rate^(1)^, however, in another recent Brazilian study with critical patients, the UR rate was similar to that of the investigation^(2)^. The variety in UR prevalence mentioned can be attributed to differences in care protocols, clinical characteristics of the populations studied, such as age, associated comorbidities (such as hypertension and diabetes), critical health status, type of surgery performed, and type of anesthesia applied, as well as in the diagnostic criteria used and the application of preventive interventions, such as the use of bladder ultrasound and individualized management strategies^(1,2,21,22)^.

The absence of urination for a certain period is related to possible UR and determines the nurse’s decision to perform insonation. In this sense, prior knowledge of the reasons that lead to a reduction in urinary activity is essential for the early performance of bladder POCUS and to provide benefits to the patient^(23)^. This study shows that the postoperative period is a key moment for the reduction of urination and the development of UR. Among the intrinsic factors related to this period are the types of surgical procedures, with emphasis on orthopedic surgeries, which have a significant incidence of UR^(20)^, in addition to the fact that surgery lasting more than four hours increases the risk of UR by up to onefold^(21)^. In an American study of patients undergoing total knee arthroplasty, it was demonstrated that certain drugs, in this case non-steroidal anti-inflammatory drugs and glycopyrrolate, administered during the intraoperative period are predictors of the risk of developing UR, with an up to fourfold increase in the risk of UR^(24)^.

Another relevant factor related to the absence of spontaneous urination is the period after removal of the DBC. An US clinical trial compared the impact of creating a protocol for early removal of the DBC in postoperative patients. An increase in UR was observed (12.4% to 26.7%, *p* = 0.003), however, there was a reduction in the rate of UTI (3.0% to 1.0%, *p* = 0.280)^(25)^. A 2020 systematic review and meta-analysis comparing early and late removal of the DBC catheter in relation to UR and UTI concluded that studies are inconclusive in comparing the inferiority of early versus late removal of the DBC catheter in relation to UR. However, early removal is superior in terms of reducing the risk of UTI^(26)^.

The creation of protocols to reduce unnecessary BC through the use of POCUS is essential. In an US study on unnecessary catheterization protocol in the postoperative period, which includes removal of the DBC within six hours postoperatively, with the participation of nurses using POCUS to measure urinary content and limit BC to volumes found on USG above 150 ml, showed a 90.0% reduction in unnecessary catheterizations, in addition to speeding up hospital discharge (4.0% to 22.0%, *p* = 0.022)^(27)^.

Despite the lack of evidence in the literature on the accuracy and training of nurses in the use of bladder POCUS, a study with five types of simulators representing different clinical situations demonstrated excellent correlation between the volumes measured by nurses and the actual volumes, although there was no stratification by volume gradations or clinical situation^(28)^.

In this regard, a US study with nurses newly trained in bladder POCUS evaluated the correlation between volumes estimated by USG and urinary volume drained by BC, and identified a strong correlation in the first insonations, with a very strong correlation from the second and third measurements^(29)^. The only Brazilian study that addressed this topic showed a very strong correlation between volumes; however, there was no stratification by volume or by the nurse’s work unit^(1)^.

In a French study, a very strong correlation was also found between the volumes analyzed and excellent accuracy according to the receiver operating characteristic (ROC) for volumes above 600 ml, corroborating the findings of the present study regarding the excellent evaluation of bladder POCUS by nurses in volumes above 500 ml^(30)^. It is important to mention that the literature is limited regarding the intrinsic factors of the practice of insonar that hinder the performance of bladder POCUS by nurses.

The implications of the results of this study for nursing practice are substantial. The use of bladder POCUS by nurses can optimize patient care, allowing for rapid and accurate interventions that significantly improve clinical outcomes. The ability to identify and quantify UR with a noninvasive and effective method can reduce the incidence of associated complications, such as UTI and prolonged hospital stay. Therefore, it is essential that nursing care protocols implement the use of bladder POCUS in different settings and contexts.

In the field of education, it is imperative that nurse training includes specific modules on the use of POCUS. The implementation of structured theoretical and practical training during undergraduate and continuing education programs can enable healthcare professionals to use this tool effectively and safely. In addition, the development of POCUS skills should be encouraged through simulators and clinical scenarios that reproduce real-life situations, ensuring that nurses are prepared to apply this knowledge in clinical practice.

For research purposes, this study opens up several opportunities for future investigations that could explore the relationship between the use of POCUS and other clinical outcomes, as well as investigate the economic impact of implementing this technology in nursing practice. However, the current research has some limitations because it is a retrospective study, which may introduce biases in data collection and interpretation. In addition, the sample consisted of patients from a single hospital center, which may limit the generalization of the results.

## CONCLUSION

The results demonstrated that the main indications for performing POCUS were absence of urination after surgical procedures, removal of the DBC, presence of RLC, and suspected RBC obstruction. The volume found by bladder POCUS performed by nurses showed a very strong correlation with the volume of urine drained by RBC or DBC, with this correlation increasing gradually as the volume visualized increased. In addition, the correlation did not vary between the different work units of the nurses. These findings reinforce the importance of including POCUS in nursing care protocols in order to promote more qualified and safer care for patients.

## References

[1] Ceratti RN, Beghetto MG (2021). Incidence of urinary retention and relations between patient’s complaint, physical examination, and bladder ultrasound. Rev Gaúcha Enferm.

[2] Lopes KR, Jorge BM, Barbosa MH, Barichello E, Nicolussi AC (2023). Use of ultrasonography in the evaluation of urinary retention in critically ill patients. Rev Latino-Am Enfermagem.

[3] Kołodziej Ł, Jurewicz A, Gębska M (2023). Nursing interventions reduce postoperative urinary retention in fast-track total hip arthroplasty: A pilot study. Adv Clin Exp Med.

[4] Bengtsen MB, Heide-Jørgensen U, Borre M, Knudsen JS, Nørgaard M (2023). Acute urinary retention in men: 21-year trends in incidence, subsequent benign prostatic hyperplasia-related treatment and mortality: A Danish population-based cohort study. Prostate.

[5] Abrams P, Cardozo L, Fall M, Griffiths D, Rosier P, Ulmsten U (2003). The standardisation of terminology in lower urinary tract function: report from the standardisation sub-committee of the International Continence Society. Urology.

[6] Billet M, Windsor TA (2019). Urinary retention. Emerg Med Clin North Am.

[7] Mavrotas J, Gandhi A, Kalogianni V, Patel V, Batura D (2022). Acute urinary retention. Br J Hosp Med (Lond).

[8] Lo E, Nicolle LE, Coffin SE, Gould C, Maragakis LL, Meddings J (2014). Strategies to Prevent catheter-associated urinary tract infections in acute care hospitals: 2014 update. Infect Control Hosp Epidemiol.

[9] Brasil. Conselho Federal de Enfermagem (2024). Resolução COFEN nº 736, de 17 de janeiro de 2024. Dispõe sobre a implementação do Processo de Enfermagem em todo contexto socioambiental onde ocorre o cuidado de enfermagem.. Diário Oficial da União [Internet].

[10] Herdman TH, Kamitsuru S, Lopes CT (2024). NANDA International Nursing Diagnoses: Definitions and Classification, 2024-2026.

[11] Moraes VM, Lucena AF, Bavaresco T, Cruz ACB, Oliveira KLR, Silva TS (2024). Desenvolvimento da Intervenção de Enfermagem “Ultrassonografia: bexiga” segundo a Nursing Interventions Classification. Acta Paul Enferm.

[12] Kalam S, Selden N, Haycock K, Lowe T, Skaggs H, Dinh VA (2023). Evaluating the effect of nursing-performed point-of-care ultrasound on septic emergency department patients. Cureus.

[13] Keita H, Diouf E, Tubach F, Brouwer T, Dahmami S, Mantz J (2005). Predictive factors of early postoperative urinary retention in the postanesthesia care unit. Anesth Analg.

[14] Conselho Regional de Enfermagem de São Paulo (2014). Parecer COREN-SP 029/2014 CT. PRCI n° 1530/2014 [Internet].

[15] Brasil (1986). Lei n° 7.498, de 25 de junho de 1986. Dispõe sobre a regulamentação do exercício da Enfermagem e dá outras providências. Diário Oficial da União [Internet].

[16] Brasil. Conselho Federal de Enfermagem (2021). Resolução COFEN nº 679/2021. Aprova a normatização da realização de Ultrassonografia à beira do leito e no ambiente pré-hospitalar por Enfermeiro. Diário Oficial da União [Internet].

[17] Santos VB, Silva WP, Apablaza MFS, Silva TV, Gimenes FRE (2024). The use of point-of-care ultrasound in nurses’ clinical practice as a foundation for patient safety. Rev Bras Enferm.

[18] Schallom M, Prentice D, Sona C, Vyers K, Arroyo C, Wessman B (2020). Accuracy of measuring bladder volumes with ultrasound and bladder scanning. Am J Crit Care.

[19] Harris PA, Taylor R, Minor BL, Elliott V, Fernandez M, O’Neal L (2019). Building an international community of software platform partners. J Biomed Inform.

[20] Jorge BM, Mazzo A, Napoleão AA, Bianchini A (2018). Scientific evidence of urinary retention diagnostic practices: scoping review. Rev Enferm UERJ.

[21] Kort NP, Bemelmans Y, Vos R, Schotanus MGM (2018). Low incidence of postoperative urinary retention with the use of a nurse-led bladder scan protocol after hip and knee arthroplasty: a retrospective cohort study. Eur J Orthop Surg Traumatol.

[22] Kołodziej Ł, Jurewicz A, Ge˛bska M (2023). Nursing interventions reduce postoperative urinary retention in fast-track total hip arthroplasty: A pilot study. Adv Clin Exp Med.

[23] Kowalik U, Plante MK (2016). Urinary retention in surgical patients. Surg Clin North Am.

[24] Bracey DN, Hegde V, Pollet AK, Johnson RM, Jennings JM, Miner TM (2021). Incidence and predictive risk factors of postoperative urinary retention after primary total knee arthroplasty. J Arthroplasty.

[25] Hu Y, Craig SJ, Rowlingson JC, Morton SP, Thomas CJ, Persinger MB (2014). Early removal of urinary catheter after surgery requiring thoracic epidural: a prospective trial. J Cardiothorac Vasc Anesth.

[26] Castelo M, Sue-Chue-Lam C, Kishibe T, Acuna SA, Baxter NN (2020). Early urinary catheter removal after rectal surgery: systematic review and meta-analysis. BJS Open.

[27] Brackmann M, Carballo E, Uppal S, Torski J, Reynolds RK, McLean K (2020). Implementation of a standardized voiding management protocol to reduce unnecessary re-catheterization A quality improvement project. Gynecol Oncol.

[28] Colombo A, Stella A, Lombardi F, Gulino S, Pregnolato S, Bonaiti S (2020). Urinary bladder test device to integrate basic ultrasound training for nurses. Ultrasound Med Biol.

[29] Frederickson M, Neitzel JJ, Miller EH, Reuter S, Graner T, Heller J (2000). The implementation of bedside bladder ultrasound technology: effects on patient and cost postoperative outcomes in tertiary care. Orthop Nurs.

[30] Daurat A, Choquet O, Bringuier S, Charbit J, Egan M, Capdevila X (2015). Diagnosis of postoperative urinary retention using a simplified ultrasound bladder measurement. Anesth Analg.

