# Supplementary material for: Assessment of bladder volume by nurses using point-of-care ultrasound: a cross-sectional study
Source: Rev Esc Enferm USP. 2025 Jul 14;59:e20240285. doi: 10.1590/1980-220X-REEUSP-2024-0285en (PMC12274074; doi:10.1590/1980-220X-REEUSP-2024-0285en)
Supplement: Translation

# Translation: Avaliação pelo enfermeiro do volume vesical por meio da ultrassonografia *point of care*: estudo transversal

**DOI:** 10.1590/1980-220X-REEUSP-2024-0285pt

**Authors:** Tatiana Fuzaro, Dejanira Aparecida Regagnin, Thales Henrique Vieira Brandão, João Paulo Victorino, Eduarda Ribeiro dos Santos, Filipe Utuari de Andrade Coelho

**Affiliations:** 1 Hospital Israelita Albert Einstein, Departamento de Pacientes Graves, São Paulo, SP, Brasil.; 2 Faculdade Israelita de Ciências da Saúde Albert Einstein, São Paulo, SP, Brasil.; 3 Universidade de São Paulo, Escola de Enfermagem, São Paulo, SP, Brasil.

**Keywords:** Enfermagem, Ultrassonografia, Retenção Urinária, Bexiga Urinária, Prática Avançada de Enfermagem

## Abstract

**Objetivos:**

Identificar os principais motivos para a realização da ultrassonografia à beira do leito *Point of Care Ultrasonography* (POCUS) vesical pelo enfermeiro e comparar o volume encontrado no POCUS com o volume de urina drenado por cateter vesical de alívio (CVA) ou cateter vesical de demora (CVD).

**Método:**

Estudo transversal realizado em unidades de terapia intensiva, semi-intensiva e clínica médico-cirúrgica com pacientes maiores de 18 que apresentavam ausência de diurese espontânea por mais de seis horas ou por mais de quatro horas após a retirada do CVD.

**Resultados:**

Incluídos 211 pacientes com principais motivos para a realização do POCUS a ausência de micção após procedimentos cirúrgicos (35,0%), retirada de CVD (35,1%), rebaixamento do nível de consciência (RNC) (13,7%) e obstrução do CVD (0,9%). A correlação entre o volume do POCUS e o volume de urina drenado foi considerada fortíssima.

**Conclusão:**

Situações como pós-operatório, retirada de CVD, RNC e obstrução do CVD levam à realização do POCUS pelo enfermeiro, com uma correlação fortíssima entre os volumes encontrados no POCUS e os drenados por CVA ou CVD.

## INTRODUÇÃO

Atualmente, a utilização da ultrassonografia (USG) pelo enfermeiro, por meio do conceito de ultrassonografia à beira do leito *point of care ultrasonography* (POCUS) tem ganhado espaço, destacando-se pela sua contribuição no manejo da retenção urinária (RU) e na avaliação do volume vesical^(1,2,3)^. A RU é uma complicação associada à distensão da bexiga, que pode causar danos ao órgão, impactar o tempo de internação e contribuir para o desenvolvimento de infecções do trato urinário (ITU)^(4)^.

De acordo com a *International Continence Society,* a RU é definida pela presença de uma bexiga dolorosa, palpável ou percutível, quando o paciente não consegue urinar^(5)^. Volumes superiores a 300 mL são frequentemente considerados como o valor mínimo necessário para identificar uma bexiga distendida, com exceção de pacientes obesos^(6)^. A incidência da RU é estimada em até 6,8 casos por 1000 pessoas-ano na população geral masculina devido à uretra mais longa e a presença da próstata, com uma razão de incidência entre homens e mulheres entre 13:1^(6,7)^. A RU é uma causa comum de morbidade, e representa mais de 30.000 admissões hospitalares por ano no sistema de saúde inglês^(7)^.

As etiologias mais frequentes da RU incluem obstrução do trato urinário por causas intrínsecas e extrínsecas, especialmente em homens, devido à hiperplasia benigna da próstata (HBP)^(6,7)^. Infecções do trato urinário (ITU), comumente causadas por *Escherichia coli*, também são significativas^(6,7)^. A origem neurogênica está associada a alterações na coordenação entre o sistema nervoso simpático e parassimpático, impactando o esvaziamento completo da bexiga^(6,7)^. Por fim, a RU iatrogênica está relacionada a questões farmacológicas, envolvendo uma variedade de medicamentos que alteram o controle vesical^(6,7)^.

É de extrema importância que a RU seja identificada rapidamente para evitar suas complicações, que incluem desde o acometimento psicológico do paciente até hematúrias devido a rápida descompressão e o desenvolvimento de infecções^(6,7)^. No que tange às complicações crônicas, estão incluídas a diminuição da contratilidade e sensibilidade da bexiga, incontinência, hidronefrose e falência renal a longo prazo^(6,7)^. O tratamento mais comum para RU é o cateterismo vesical (CV), tanto de alívio (CVA) quanto de demora (CVD), é importante ressaltar que cerca de 80% das ITU em pacientes internados estão relacionadas ao CV^(6,7,8)^.

Nesse contexto, a avaliação vesical pelo enfermeiro é de suma importância para obter informações que possam servir de fundamentos para ações baseadas no processo de enfermagem^(9,10)^. O enfermeiro, ao se deparar com o diagnóstico de enfermagem de eliminação urinária prejudicada, conforme proposto pela NANDA *International*, é responsável por avaliar dados clínicos do paciente e propor intervenções de enfermagem relevantes que promovam melhora ou estabilização da situação encontrada^(10,11)^. Recentemente, foi desenvolvida a intervenção de enfermagem “ultrassonografia de bexiga” segundo a Classificação das Intervenções de Enfermagem (NIC), que estará presente na próxima edição da NIC, visto que contempla itens destinados à prática do POCUS na avaliação vesical pelo enfermeiro^(11)^.

A USG é atualmente o método não invasivo mais eficaz como ferramenta semiológica auxiliar, de maneira a fornecer informações imediatas à beira do leito para confirmar os achados do exame físico, o que permite maior agilidade na tomada de decisão^(12)^. No caso de RU, a USG é um método confiável, com sensibilidade de 97%, especificidade de 91% e acurácia de 94%^(13)^.

Cabe enfatizar que o parecer do Conselho Regional de Enfermagem de São Paulo (COREN-SP) COREN-SP 029/2014 – CT PRCI n° 1530/2014, junto da Lei do Exercício da Profissão de Enfermagem (Lei nº 7.498, de 25 de junho de 1986), assegura a autonomia para a utilização da USG com objetivo de mensuração do volume vesical realizada por enfermeiro capacitado^(14,15)^. Ressalta-se ainda que, em 2021, o Conselho Federal de Enfermagem (COFEN) emitiu o parecer COFEN nº 679/2021, que normatizou a realização do POCUS à beira do leito^(16)^. Contudo, a realização de laudos de exames e diagnósticos nosocomiais não cabe ao profissional enfermeiro, visto que é uma atividade privativa do profissional médico^(14,16)^.

A utilização do POCUS pelo enfermeiro no Brasil é recente e tem recebido destaque devido à sua aplicabilidade nas diversas áreas do cuidado pela enfermagem^(17)^. No entanto, a literatura ainda é escassa no que se refere à USG e à prática do enfermeiro na mensuração do volume vesical por meio do POCUS. Em um único estudo brasileiro com pacientes adultos em unidades de internação, foi evidenciada uma forte correlação entre o volume de urina drenado por CVA e o volume vesical encontrado pelo enfermeiro na USG antes do CVA^(1)^. Outro estudo brasileiro com pacientes críticos evidenciou o auxílio da USG na detecção da RU pelo enfermeiro, mas não apresentou informações aprofundadas sobre a prática do POCUS vesical, de modo a focar principalmente nos fatores relacionados ao desenvolvimento da RU^(2)^.

Diante do exposto, podemos concluir que o uso da USG para mensuração do volume vesical realizado pelo enfermeiro à beira do leito é uma avaliação rápida, não invasiva e que propicia uma tomada de decisão mais acurada. Portanto, diante da escassez de estudos nacionais que contextualizem sobre as questões relacionadas a realização do POCUS vesical na prática do enfermeiro, faz-se necessário aprofundamento nesta temática. Assim, o objetivo do presente estudo é identificar os principais motivos para a realização do POCUS vesical pelo enfermeiro e comparar o volume identificado no POCUS com o volume de urina drenado por CVA ou CVD.

## MÉTODO

### Tipo do Estudo

Trata-se de um estudo transversal retrospectivo, elaborado seguindo as recomendações do checklist STROBE para garantir maior transparência e consistência na apresentação dos dados.

### Local

O estudo foi realizado em um hospital de grande porte, localizado na cidade de São Paulo, SP, e incluiu três unidades destinadas ao atendimento de pacientes adultos: a unidade de terapia intensiva, com 42 leitos de caráter geral; a unidade de cuidados semi-intensivos, com 50 leitos; e as unidades de clínica médico-cirúrgica, que possuem cerca de 200 leitos.

### Capacitação dos Enfermeiros em Pocus Vesical

Na referida instituição, a partir de 2015, foi desenvolvido um protocolo institucional direcionado para mensuração do volume vesical por meio do POCUS realizado pelo enfermeiro. Desde então, os enfermeiros são capacitados para o uso dessa ferramenta após sua admissão na instituição.

A capacitação institucional dos enfermeiros para realização do POCUS vesical consiste em uma explanação teórica com duração de duas horas, a qual aborda aspectos teóricos essenciais para entendimento e manipulação do equipamento da USG, além do conteúdo específico do POCUS. Ainda como conteúdo deste momento há uma breve revisão anatômica da bexiga com seus planos anatômicos para sua visualização e a técnica de mensuração do volume vesical. Importante mencionar que o grupo de treinamento é composto por 5 a 10 enfermeiros, com o objetivo de equilibrar adequadamente o número de participantes em relação ao número de instrutores, bem como à disponibilidade do equipamento de USG e de voluntários para a prática.

Após o momento teórico é realizada uma parte prática estruturada em atividade *hands on*, com duração de duas horas, para o desenvolvimento de habilidades no manuseio do equipamento de USG e na aplicação da técnica do POCUS vesical em voluntários. Nesta fase, os profissionais são acompanhados por uma enfermeira de referência em POCUS na instituição, quem também ministra a parte teórica.

Durante o tempo de treinamento prático, são utilizados dois voluntários da própria instituição, geralmente monitores da graduação em enfermagem, sendo estimulados a ingerirem água periodicamente com o intuito de manter bexiga com volume de urina para a realização dos exames pelos enfermeiros. Ressalta-se que os profissionais são orientados a realizarem de cinco a dez insonações nos voluntários, acompanhados da instrutora mencionada, e após este momento, são submetidos a uma avaliação prática. Esta avaliação consiste na realização da técnica do POCUS vesical em um dos voluntários, podendo ser considerado apto ou não apto pela instrutora, com base em um *checklist* desenvolvido pela própria instituição que contém os itens fundamentais da respectiva técnica. A aprovação do profissional este momento da capacitação ocorre mediante somatórias de acertos igual ou superior a 80% dos itens presentes no *checklist*. Caso seja considerado não apto, o profissional é convocado para novo treinamento com turmas futuras e não está liberado para esta prática em sua unidade de trabalho até sua aprovação. Importante ressaltar que as unidades escolhidas para coleta de dados do presente estudo, no período selecionado, contavam com enfermeiros com mais de seis meses de experiência em POCUS vesical.

De acordo com o protocolo institucional de POCUS vesical, em situações em que o paciente não apresenta diurese espontânea por mais de seis horas ou por mais de quatro horas após a retirada do CVD, o enfermeiro deve realizar um exame físico para avaliar a presença de globo vesical palpável, sinais de desconforto ou distensão abdominal, presença de vontade de urinar ou ainda agitação e sudorese. Após esta avaliação, o enfermeiro deverá realizar o POCUS vesical com o intuito de mensurar a quantidade de volume da bexiga. A depender do volume encontrado, o protocolo institucional sugere manobras de esvaziamento, como ficar em pé ao lado do leito, realizar a manobra de Valsalva, aplicar calor na região supra púbica, promover o som de água corrente e sentar-se no vaso sanitário. Caso estas ações não sejam efetivas ou não indicadas pelo quadro clínico, e o volume obtido no POCUS vesical for maior ou igual a 400 ml, ou acima de 200 ml acompanhado de sintomas de desconforto, o médico é acionado para discussão da necessidade da CVA. A CVD está indicada para volumes maiores que 1000 ml encontrado no POCUS vesical e com episódios prévios de RU^(18)^. A definição utilizada neste estudo para caracterizar a RU foi volume de urina acima de 500 ml encontrado no POCUS vesical^(2)^.

Os aparelhos de ultrassom utilizados na instituição são equipamentos SonoSite M-Turbo®, registrados na Agência Nacional de Vigilância Sanitária sob o Nº 80022060089 e licenciados para uso profissional e para o atendimento da população. Estes equipamentos estão configurados para avaliar imagens utilizando sondas do tipo convexas, lineares e setoriais. Cada unidade mencionada anteriormente (UTI, semi-intensiva e clínica médico-cirúrgica) dispõem deste equipamento para utilização dos enfermeiros. Para a realização da técnica de mensuração do volume vesical utilizada na instituição, o paciente deve ser posicionado em decúbito dorsal horizontal. Em seguida, posiciona-se o transdutor convexo na região suprapúbica no plano transverso. Nesse momento, ao insonar a bexiga, realiza-se o cálculo do comprimento do diâmetro latero-lateral da bexiga. Posteriormente, altera-se a posição do transdutor, mantendo-o no local anatômico, para a visualização no plano longitudinal. Nessa etapa, a insonação da bexiga deve incluir o cálculo dos comprimentos dos diâmetros ântero-posterior e crânio-caudal. O equipamento utilizado na instituição realiza automaticamente o cálculo do volume vesical.

### Amostra e Critérios de Seleção

A amostra foi por conveniência, composta por prontuários de pacientes maiores de 18 anos, avaliados com POCUS vesical pelo enfermeiro, que não apresentaram diurese espontânea por mais de seis horas ou após a retirada do CVD por mais de quatro horas e tiveram como ação após o procedimento a introdução do CVA ou CVD, durante sua internação nas unidades mencionadas, no período de janeiro de 2017 a dezembro de 2018. Após a identificação dos pacientes por meio de um banco de dados, foram encontrados 395 indivíduos elegíveis. Todos os pacientes receberam contato telefônico e, após a apresentação do estudo, os pesquisadores enviaram o Termo de Consentimento Livre e Esclarecido (TCLE) pela plataforma *Google Forms*. Desses, 25 recusaram a participação e 99 apresentaram registros incompletos nos prontuários, o que resultou na inclusão de 211 pacientes no estudo. Ressalta-se que houve contato com todos os pacientes elegíveis.

### Variáveis do Estudo

As variáveis coletadas incluíram informações demográficas (sexo e idade), características clínicas (diagnóstico de internação, antecedentes clínicos, exame físico, e potenciais medicamentos em uso que interferem no esvaziamento vesical) e dados relacionados à realização do POCUS (tempo de ausência de micção, motivo da realização da USG, volume de urina encontrado na USG, condutas após a realização da USG e o volume de urina drenado posterior por CVA ou CVD).

### Coleta de Dados

A coleta de dados foi realizada pelos autores por meio da revisão dos prontuários eletrônicos, após a autorização dos participantes mediante a assinatura do TCLE. A identificação dos prontuários ocorreu em janeiro de 2020, o contato com os pacientes foi realizado entre março e abril de 2020, e a coleta de dados por meio da revisão dos prontuários aconteceu entre maio e junho do mesmo ano.

### Análise e Tratamento dos Dados

Os dados foram inseridos na plataforma *Research Electronic Data Capture*® (REDCap®)^(19)^ e importados para planilha eletrônica do programa *Microsoft Excel* ® 2007. Para as análises, foi usado o software *Statistical Package for the Social Science* (SPSS), versão 26. A análise descritiva foi realizada por meio de frequências absolutas e relativas para variáveis qualitativas, enquanto para as quantitativas, primeiramente foi testada a normalidade pelo teste de *Shapiro-Wilk.* Após a verificação da normalidade, foram calculados a média e o desvio padrão. Em relação a análise inferencial, foram utilizados os testes de qui-quadrado e *Fisher* para as variáveis qualitativas e o teste T de *Student* para as quantitativas.

Para a análise da comparação entre o volume vesical observado na POCUS pelo enfermeiro com o posterior volume encontrado de urina na CVA ou CVD foi utilizado o coeficiente de correlação de *Person*. O nível de significância adotado para a análise foi de *p* ≤ 0,005.

### Aspectos Éticos

A presente pesquisa esteve de acordo com a resolução nº 466/2012, sendo submetida e aprovada pelo Comitê de Ética em Pesquisa da referida instituição, sob número de aprovação 4.533.418 e CAEE: 40058020700000071.

## RESULTADOS

Foram incluídos um total de 211 pacientes, que tiveram a realização de 211 POCUS vesical pelo enfermeiro (um por paciente). Na Tabela 1 está a caracterização dos pacientes submetidos a realização do POCUS vesical pelo enfermeiro de acordo com a presença ou não da RU. A RU esteve presente em 119(56,3%) dos pacientes, dos quais mais da metade eram do sexo masculino, com média de idade em 65,1 ± 20,2 anos. A hipertensão arterial sistêmica (HAS) foi o antecedente clínico mais comum, e o diagnóstico de internação de pós-operatório imediato foi mais frequente no grupo com RU. Entre os medicamentos em uso com potencial para alteração da frequência urinária, destacam-se os opioides nos pacientes com RU.

**Tabela 1:** Caracterização dos pacientes que foram submetidos a realização do POCUS vesical pelo enfermeiro de acordo com a presença ou não da RU – São Paulo, SP, Brasil, 2020.

Variável	Total n = 211 (%)	Sem RU n = 92 (%)	Com RU n = 119 (%)	Valor de *p*
Sexo				
Masculino	123 (58,3)	51 (55,4)	72 (60,5)	0,459^a^
Feminino	88 (41,7)	41 (44,6)	47 (39,5)	
Antecedentes clínicos				
HAS	81 (38,4)	41 (44,6)	40 (33,6)	0,105^a^
DM	30 (14,2)	14 (15,2)	16 (13,4)	0,715^a^
DPOC	14 (6,6)	9 (9,8)	5 (4,2)	0,106^a^
IC	14 (6,6)	9 (9,8)	5 (4,2)	0,106^a^
DRC	8 (3,8)	4 (4,3)	4 (3,4)	0,710^a^
Diagnóstico de internação				
Pós-operatório	103 (48,8)	35 (38,0)	68 (57,1)	0,006^a^
Neurológico	42 (19,9)	24 (26,1)	18 (15,1)	0,048^a^
Respiratório	25 (11,8)	15 (16,3)	10 (8,4)	0,078^a^
Infeccioso	22 (10,4)	9 (9,8)	13 (10,9)	0,788^a^
Cardiovascular	13 (6,2)	4 (4,3)	9 (7,6)	0,335^a^
Urológico	12 (5,7)	7 (7,6)	5 (4,2)	0,289^a^
Gastrointestinal	7 (3,3)	3 (3,3)	4 (3,3)	0,748^a^
Classe farmacológica				
Opioide	63 (29,9)	18 (19,6)	45 (37,8)	0,004^a^
Antipsicótico	38 (18,0)	18 (19,6)	20 (16,8)	0,605^a^
Analgésico narcótico	36 (17,1)	16 (17,4)	20 (16,8)	0,911^a^
Antiespasmódico	27 (12,8)	14 (15,2)	13 (10,9)	0,355^a^
Antidepressivo	14 (6,6)	6 (6,5)	8 (6,7)	0,954^a^
Antiparkinsoniano	11 (5,2)	5 (5,4)	6 (5,0)	> 0,999^b^
Óbito	49 (23,2)	26 (28,3)	23 (19,3)	0,128^a^

RU: retenção urinária, HAS: hipertensão arterial sistêmica; DM: Diabetes Mellitus; DPOC: doença pulmonar obstrutiva crônica; IC: insuficiência cardíaca; DRC: doença renal crônica; UTI: unidade de tratamento intensivo, a: teste do quiquadrado, b: teste exato de *Fisher*.

As informações relacionadas à realização do POCUS vesical e envolvidas na micção de acordo com a presença ou não de RU estão contidas na Tabela 2. A unidade que mais realizou o POCUS vesical foi a unidade de clínica médico-cirúrgica. A presença de globo vesical palpável mais frequente foi no grupo com RU, e o motivo predominante para a ausência de micção que levou à realização do POCUS pelo enfermeiro esteve associado com a realização de procedimentos cirúrgicos. O volume vesical encontrado no POCUS foi significativamente superior nos pacientes com RU. A principal ação após a realização do POCUS vesical foi a introdução do CVA, e o volume de urina drenado pelo CVA ou CVD após o POCUS foi maior no grupo com RU.

**Tabela 2:** Informações relacionadas à realização do POCUS vesical e envolvidas na micção de acordo com a presença ou não de RU – São Paulo, SP, Brasil, 2020.

Variável	Total (n = 211)	Sem RU (n = 92)	Com RU (n = 119)	Valor de *p*
Unidade de realização do POCUS				
Clínica médico-cirúrgica	142 (67,3)	56 (60,9)	86 (72,3)	0,080^a^
Semi-intensiva	36 (17,1)	19 (20,7)	17 (14,3)	0,223^a^
UTI	33 (15,6)	17 (18,5)	16 (13,4)	0,318^a^
Tempo sem urinar (horas)				
Média ± DP	7,5 ± 2,5	7,6 ± 2,4	7,4 ± 2,5	0,586^b^
Globo vesical palpável n (%)	113 (56,3)	38 (41,3)	75 (63,0)	0,002^a^
Causas da ausência de micção				
Procedimento cirúrgico n (%)	75 (35,5)	23 (25,0)	52 (43,7)	0,005^a^
Retirada de CVD n (%)	74 (35,1)	40 (43,5)	34 (28,6)	0,024^a^
RNC n (%)	29 (13,7)	19 (20,7)	10 (8,4)	0,010^a^
Obstrução de CVD n (%)	2 (0,9)	1 (1,1)	1 (0,8)	> 0,999^c^
Volume verificado no POCUS				
Média ± DP	563,3 ± 251,8	345,0 ± 107,5	732,1 ± 195,0	< 0,001^b^
Ação após o POCUS				
CVA n (%)	190 (90,0)	81 (88,0)	109 (91,5)	0,544^a^
CVD n (%)	21 (10,0)	10 (10,9)	11 (9,2)	0,696^a^
Volume drenado por CVA ou CVD após POCUS				
Média ± DP	593,1 ± 290,3	395,0 ± 167,3	746,3 ± 272,4	< 0,001^b^

RU; retenção urinária, POCUS: *point of care ultrasonography*, UTI: unidade de tratamento intensivo, CVD: cateter vesical de demora, RNC: rebaixamento do nível de consciência, CVA cateter vesical de alívio, a: teste do quiquadrado, b: teste T de *Student*, c; teste exato de *Fisher*.

Na Figura 1, é possível verificar a correlação entre os volumes estimados pelo POCUS realizado pelo enfermeiro e o volume de urina verificado posteriormente através da introdução do CVA ou CVD. Na Figura 1A, observa-se a correlação entre o volume do POCUS até 200 ml e o volume pelo CVA ou CVD (r = 0,759, *p* < 0,001). A Figura 1B evidencia a correlação entre o volume do POCUS de 200 a 500 ml e o volume pelo CVA ou CVD (r = 0,836, *p* < 0,001). A Figura 1C demonstra a correlação entre o volume do POCUS acima de 500 ml e o volume pelo CVA ou CVD (r = 0,922, *p* < 0,001). Por fim, a Figura 1D ilustra a correlação entre o volume do POCUS, independente da quantidade de ml encontrados, e o volume pelo CVA ou CVD (r = 0,958, *p* < 0,001).

**Figura 1:** Correlação entre os volumes do POCUS e o volume de urina verificado na posterior introdução do CVA ou CVD – São Paulo, SP, Brasil, 2020.

POCUS: *point of care ultrasonography*, CVA cateter vesical de alívio, CVD: cateter vesical de demora, ml: mililitros.

A correlação entre os volumes estimados no POCUS vesical realizado pelo enfermeiro, de acordo com a unidade de internação, e o volume de urina verificado posteriormente através da introdução do CVA ou CVD está na Figura 2. Na Figura 2A, observa-se a correlação entre o volume do POCUS vesical encontrado por enfermeiros da clínica médico-cirúrgica e o volume de urina drenado por CVA ou CVD (r = 0,959, *p* < 0,001). A Figura 2B evidencia a correlação entre o volume do POCUS vesical mensurado pelos enfermeiros de cuidados semi- intensivos e o volume drenado por CVA ou CVD (r = 0,985, *p* < 0,001). A Figura 2C demonstra a correlação entre o volume do POCUS vesical insonado por enfermeiros da UTI e o volume drenado por CVA ou CVD (r = 0,940, *p* < 0,001).

**Figura 2:** Correlação entre os volumes do POCUS pelo enfermeiro, de acordo com a unidade, e o volume de urina verificado em posterior introdução do CVA ou CVD. Na Figura 1A está a correlação entre o volume do POCUS vesical encontrado por enfermeiros da clínica médico-cirúrgica, na Figura 1B pelos enfermeiros da semi-intensiva e na Figura 1C pelos enfermeiros da UTI – São Paulo, SP, Brasil, 2020.

POCUS: *point of care ultrasonography*, CVA cateter vesical de alívio, CVD: cateter vesical de demora, ml: mililitros.

## DISCUSSÃO

No presente estudo é possível observar um perfil de pacientes idosos, que em sua maioria foram submetidos a algum tipo de procedimento cirúrgico com necessidade de avaliação do enfermeiro por não apresentarem diurese, principalmente após retirada de CVD. A correlação entre o volume encontrado no POCUS vesical pelo enfermeiro e o volume de urina drenado por CVA ou CVD, de uma forma geral, foi significativamente forte, uma vez que se modifica gradativamente pela quantidade de volume encontrada no POCUS, e não apresenta diferença pelo local de trabalho do enfermeiro.

Em relação à prevalência de RU, mais da metade dos pacientes analisados apresentaram esta alteração. Em revisão de escopo brasileira, mostrou que dentre as principais práticas utilizadas para o diagnóstico da RU, destaca-se o uso da USG para tomada de decisão frente ao CV^(20)^. Em estudo holandês com pacientes em pós-operatório de cirurgia de artroplastia de quadril e joelho a frequência de RU foi significativamente inferior à do presente estudo^(21)^. No entanto, estudo polonês com pacientes no mesmo contexto cirúrgico, a prevalência encontrada foi similar a nossa pesquisa^(22)^. No cenário nacional, estudo com pacientes de clínica médico-cirúrgica apresentou uma taxa de RU inferior^(1)^, entretanto, em outro estudo brasileiro recente, com pacientes críticos, a taxa de RU encontrada foi similar à presente investigação^(2)^. A variedade entre as prevalências de RU mencionadas pode ser atribuída a diferenças nos protocolos assistenciais, nas características clínicas das populações estudadas, como idade, comorbidades associadas (como hipertensão e diabetes), estado crítico de saúde, tipo de cirurgia realizada e tipo de anestesia aplicada, bem como nos critérios diagnósticos utilizados e na aplicação de intervenções preventivas, como o uso de ultrassonografia vesical e estratégias de manejo individualizado^(1,2,21,22)^.

A ausência de micção por um determinado período tem relação com uma possível RU, e determina a tomada de decisão da insonação pelo enfermeiro. Nesse sentido, o conhecimento prévio dos motivos que propiciam uma redução da atividade miccional é essencial para realização precoce do POCUS vesical e que propiciem benefícios ao paciente^(23)^. É visto no presente estudo que o período pós-operatório é um momento preponderante para redução da micção e ao desenvolvimento da RU. Entre os fatores intrínsecos relacionados a este período estão os tipos de procedimentos cirúrgicos, com destaque a cirurgias ortopédicas que apresentam uma significativa frequência de incidência de RU^(20)^, além de que o tempo de cirurgia acima de quatro horas chega a duplicar o risco de RU^(21)^. Em um estudo americano com pacientes submetidos à artroplastia total de joelho, foi demonstrado que determinados fármacos, no caso anti-inflamatórios não esteroidais e glicopirrolato, administrados no período intraoperatório são preditores no risco de desenvolvimento de RU, com aumento no risco de RU em até quatro vezes^(24)^.

Outro fator relevante relacionado à ausência de micção espontânea é o período após a retirada do CVD. Um ensaio clínico americano comparou o impacto da criação de um protocolo para remoção precoce do CVD em pacientes pós-operatórios. Observou-se um aumento da RU (12,4% para 26,7%, *p* = 0,003), entretanto, houve uma redução na taxa de ITU (3,0% para 1,0%, *p* = 0,280)^(25)^. Em revisão sistemática e metanálise de 2020, que comparou a retirada precoce do CVD com a tardia, referente a RU e ITU, estableceu que os estudos são inconclusivos para comparar a inferioridade da retirada precoce versus a tardia do CVD em relação ao RU. No entanto, a remoção precoce é superior em termos de redução para o risco de ITU^(26)^.

A criação de protocolos para diminuir a CV desnecessária, através do uso do POCUS é fundamental. Em estudo americano sobre protocolo de cateterização desnecessária em período de pós-operatório, que inclui a retirada do CVD até seis horas do pós-operatório, com a participação do enfermeiro através do POCUS para mensurar o conteúdo urinário e limitar a CV apenas para volume encontrados no USG acima de 150 ml, demonstrou uma redução de 90,0% de cateterizações desnecessárias, além de acelerar o fluxo de alta hospitalar (4,0% para 22,0%, *p* = 0,022)^(27)^.

Apesar da escassez de evidências na literatura sobre a acurácia e a capacitação do enfermeiro no uso do POCUS vesical, um estudo com cinco tipos de simuladores representando diferentes situações clínicas demonstrou excelente correlação entre os volumes mensurados pelo enfermeiro e os volumes reais, embora não tenha havido estratificação por gradações de volume ou por situação clínica^(28)^.

Nesse sentido, um estudo norte-americano com enfermeiros recém-habilitados em POCUS vesical avaliou a correlação entre os volumes estimados por USG e o volume urinário drenado por CV, e identificou correlação forte nas primeiras insonações, com correlação fortíssima a partir da segunda e terceira mensuração^(29)^. O único estudo brasileiro que abordou sobre esta temática, evidenciou uma correlação fortíssima entre os volumes, entretanto, não houve estratificação pela quantidade de volumes ou pela unidade de trabalho do enfermeiro^(1)^.

Em estudo francês, também foi encontrado uma correlação fortíssima entre os volumes analisados e uma excelente acurácia de acordo com a *receiver operating characteristic* (ROC) para volume acima de 600 ml, de forma a corroborar com os achados do presente estudo em relação excelente avaliação do POCUS vesical pelo enfermeiro em volume acima de 500 ml^(30)^. É relevante mencionar que a literatura é limitada quantos aos fatores intrínsecos à prática do insonar que dificultam a realização do POCUS vesical pelo enfermeiro.

As implicações dos resultados deste estudo para a prática de enfermagem são substanciais. A utilização do POCUS vesical por enfermeiros pode otimizar o cuidado com os pacientes, permitindo intervenções rápidas e precisas que melhoram significativamente os desfechos clínicos. A capacidade de identificar e quantificar a RU com método não invasivo e eficaz pode reduzir a incidência de complicações associadas, como ITU e prolongamento da internação hospitalar. Portanto, é fundamental que os protocolos assistenciais de enfermagem implementem a utilização do POCUS vesical em diferentes cenários e contextos.

No âmbito do ensino, é imperativo que a formação de enfermeiros inclua módulos específicos sobre a utilização do POCUS. A implementação de treinamentos teórico-práticos estruturados durante a graduação e em programas de educação continuada pode capacitar os profissionais de saúde a utilizarem essa ferramenta de maneira eficaz e segura. Além disso, o desenvolvimento de habilidades em POCUS deve ser incentivado através de simuladores e cenários clínicos que reproduzam situações reais, garantindo que os enfermeiros estejam preparados para aplicar esses conhecimentos na prática clínica.

Para a pesquisa, este estudo abre diversas oportunidades para investigações futuras que poderiam explorar a relação entre o uso do POCUS e outros desfechos clínicos, além de investigar o impacto econômico da implementação dessa tecnologia na prática de enfermagem. No entanto, a vigente pesquisa apresenta algumas limitações por tratar-se de um estudo retrospectivo, o que pode introduzir vieses na coleta e interpretação dos dados. Além disso, a amostra foi composta por pacientes de um único centro hospitalar, o que pode limitar a generalização dos resultados.

## CONCLUSÃO

Os resultados demonstraram que as principais indicações para a realização do POCUS foram a ausência de micção após procedimentos cirúrgicos, retirada do CVD, presença do RNC e suspeita de obstrução do CVD. O volume encontrado pelo POCUS vesical realizado pelo enfermeiro apresentou uma correlação fortíssima com o volume de urina drenado por CVA ou CVD, e que ainda essa correlação aumenta gradativamente conforme o volume. Além disso, a correlação não variou entre as diferentes unidades de trabalho dos enfermeiros. Esses achados reforçam a importância da inclusão do POCUS nos protocolos assistenciais de enfermagem, de forma a promover uma assistência mais qualificada e segura aos pacientes.

# Translation: Evaluación por parte del personal de enfermería del volumen vesical mediante ecografía en el punto de atención: estudio transversal

**DOI:** 10.1590/1980-220X-REEUSP-2024-0285es

**Authors:** Tatiana Fuzaro, Dejanira Aparecida Regagnin, Thales Henrique Vieira Brandão, João Paulo Victorino, Eduarda Ribeiro dos Santos, Filipe Utuari de Andrade Coelho

**Affiliations:** 1 Hospital Israelita Albert Einstein, Departamento de Pacientes Graves, São Paulo, SP, Brasil.; 2 Faculdade Israelita de Ciências da Saúde Albert Einstein, São Paulo, SP, Brasil.; 3 Universidade de São Paulo, Escola de Enfermagem, São Paulo, SP, Brasil.

**Keywords:** Enfermería, Ultrasonografía, Retención Urinaria, Vejiga Urinaria, Enfermería de Práctica Avanzada

## Abstract

**Objetivos:**

Identificar los principales motivos para la realización de ecografías vesicales a pie de cama *Point of Care Ultrasonography* (POCUS) por parte del personal de enfermería y comparar el volumen encontrado en la POCUS con el volumen de orina drenado por catéter vesical de alivio (CVA) o catéter vesical de demora (CVD).

**Método:**

Estudio transversal realizado en unidades de terapia intensiva, semiintensiva y médico-quirúrgica con pacientes mayores de 18 años que presentaban ausencia de diuresis espontánea durante más de seis horas o durante más de cuatro horas tras la retirada del CVD.

**Resultados:**

Se incluyeron 211 pacientes con motivos principales para la realización del POCUS: ausencia de micción tras procedimientos quirúrgicos (35,0 %), retirada del CVD (35,1 %), disminución del nivel de conciencia (RNC) (13,7 %) y obstrucción del CVD (0,9 %). La correlación entre el volumen del POCUS y el volumen de orina drenada se consideró muy fuerte.

**Conclusión:**

Situaciones como el postoperatorio, la retirada de la DVC, la RNC y la obstrucción de la DVC llevan a la realización del POCUS por parte del enfermero, con una correlación muy fuerte entre los volúmenes encontrados en el POCUS y los drenados por la CVA o la DVC.

## INTRODUCCIÓN

El uso de la ecografía (EG) por parte del personal de enfermería, a través del concepto actual de ecografía a pie de cama *(point of care ultrasonography*, POCUS), ha ganado terreno, destacándose por su contribución al manejo de la retención urinaria (RU) y a la evaluación del volumen vesical^(1,2,3)^. La RU es una complicación asociada a la distensión de la vejiga, que puede causar daños al órgano, afectar al tiempo de hospitalización y contribuir al desarrollo de infecciones del tracto urinario (ITU)^(4)^.

De acuerdo con la *International Continence Society,* la RU se define por la presencia de una vejiga dolorosa, palpable o percutible, cuando el paciente no puede orinar^(5)^. Los volúmenes superiores a 300 ml se consideran a menudo como el valor mínimo necesario para identificar una vejiga distendida, con excepción de los pacientes obesos^(6)^. La incidencia de la RU se estima en hasta 6,8 casos por cada 1000 personas-año en la población masculina general debido a la uretra más larga y a la presencia de la próstata, con una razón de incidencia entre hombres y mujeres de 13:1^(6,7)^. La RU es una causa común de morbilidad y representa más de 30 000 ingresos hospitalarios al año en el sistema sanitario inglés^(7)^.

Las etiologías más frecuentes de la RU incluyen la obstrucción del tracto urinario por causas intrínsecas y extrínsecas, especialmente en hombres, debido a la hiperplasia benigna de próstata (HBP)^(6,7)^. Las infecciones del tracto urinario (ITU), comúnmente causadas por *Escherichia coli*, también son significativas^(6,7)^. El origen neurogénico está asociado a alteraciones en la coordinación entre el sistema nervioso simpático y parasimpático, lo que afecta al vaciado completo de la vejiga^(6,7)^. Por último, la RU iatrogénica está relacionada con cuestiones farmacológicas, que implican una variedad de medicamentos que alteran el control vesical^(6,7)^.

Es sumamente importante identificar rápidamente la RU para evitar sus complicaciones, que incluyen desde el impacto psicológico del paciente hasta hematurias debido a la rápida descompresión y el desarrollo de infecciones^(6,7)^. Entre las complicaciones crónicas se incluyen la disminución de la contractilidad y la sensibilidad de la vejiga, la incontinencia, la hidronefrosis y la insuficiencia renal a largo plazo^(6,7)^. El tratamiento más común para la RU es el cateterismo vesical (CV), tanto de alivio (CVA) como de demora (CVD). Es importante destacar que alrededor del 80 % de las ITU en pacientes hospitalizados están relacionadas con el CV^(6,7,8)^.

En este contexto, la evaluación vesical por parte del enfermero es de suma importancia para obtener información que pueda servir de base para acciones basadas en el proceso de enfermería^(9,10)^. El enfermero, al enfrentarse al diagnóstico de enfermería de eliminación urinaria alterada, según lo propuesto por NANDA *International*, es responsable de evaluar los datos clínicos del paciente y proponer intervenciones de enfermería relevantes que promuevan la mejora o estabilización de la situación encontrada^(10,11)^. Recientemente, se ha desarrollado la intervención de enfermería «ecografía de vejiga» según la Clasificación de Intervenciones de Enfermería (NIC), que estará presente en la próxima edición de la NIC, ya que incluye elementos destinados a la práctica del POCUS en la evaluación vesical por parte del enfermero^(11)^.

La EG es actualmente el método no invasivo más eficaz como herramienta semiológica auxiliar, ya que proporciona información inmediata a pie de cama para confirmar los hallazgos de la exploración física, lo que permite una mayor agilidad en la toma de decisiones^(12)^. En el caso de la RU, la EG es un método fiable, con una sensibilidad del 97 %, una especificidad del 91 % y una precisión del 94 %^(13)^.

Cabe destacar que el dictamen del Consejo Regional de Enfermería de São Paulo (COREN-SP) COREN-SP 029/2014 – CT PRCI n.º 1530/2014, junto con la Ley del Ejercicio de la Profesión de Enfermería (Ley n.º 7.498, de 25 de junio de 1986), garantiza la autonomía para el uso de la EG con el objetivo de medir el volumen vesical realizado por un enfermero capacitado^(14,15)^. Cabe destacar que, en 2021, el Consejo Federal de Enfermería (COFEN) emitió el dictamen COFEN n.º 679/2021, que reguló la realización del POCUS a pie de cama^(16)^. Sin embargo, la realización de informes de exámenes y diagnósticos nosocomiales no corresponde al profesional de enfermería, ya que es una actividad exclusiva del profesional médico^(14,16)^.

El uso del POCUS por parte de enfermeros en Brasil es reciente y ha recibido atención debido a su aplicabilidad en diversas áreas de la atención de enfermería^(17)^. Sin embargo, la literatura aún es escasa en lo que se refiere a la EG y la práctica de los enfermeros en la medición del volumen vesical mediante el POCUS. En un único estudio brasileño con pacientes adultos en unidades de hospitalización, se evidenció una fuerte correlación entre el volumen de orina drenado por la CVA y el volumen vesical encontrado por el enfermero en la EG antes de la CVA^(1)^. Otro estudio brasileño con pacientes críticos evidenció la ayuda de la EG en la detección de la RU por parte del enfermero, pero no presentó información detallada sobre la práctica del POCUS vesical, centrándose principalmente en los factores relacionados con el desarrollo de la RU^(2)^.

Ante lo expuesto, podemos concluir que el uso de la EG para la medición del volumen vesical realizada por el enfermero a pie de cama es una evaluación rápida, no invasiva y que permite una toma de decisión más precisa. Por lo tanto, ante la escasez de estudios nacionales que contextualicen las cuestiones relacionadas con la realización de la POCUS vesical en la práctica del enfermero, es necesario profundizar en este tema. Así, el objetivo del presente estudio es identificar las principales razones para la realización de la POCUS vesical por parte del enfermero y comparar el volumen identificado en la POCUS con el volumen de orina drenado por CVA o CVD.

## MÉTODO

### Tipo de Estudio

Se trata de un estudio transversal retrospectivo, elaborado siguiendo las recomendaciones de la lista de verificación STROBE para garantizar una mayor transparencia y coherencia en la presentación de los datos.

### Lugar

El estudio se llevó a cabo en un hospital de gran tamaño, situado en la ciudad de São Paulo, SP, e incluyó tres unidades destinadas a la atención de pacientes adultos: la unidad de terapia intensiva, con 42 camas de carácter general; la unidad de cuidados semi-intensivos, con 50 camas; y las unidades de clínica médico-quirúrgica, que cuentan con unas 200 camas.

### Capacitación de Enfermeros en Pocus Vesical

En dicha institución, a partir de 2015, se desarrolló un protocolo institucional dirigido a la medición del volumen vesical mediante POCUS realizado por enfermeros. Desde entonces, los enfermeros reciben capacitación para el uso de esta herramienta tras su admisión en la institución.

La capacitación institucional de los enfermeros para la realización de la ecografía vesical consiste en una explicación teórica de dos horas de duración, que aborda aspectos teóricos esenciales para la comprensión y el manejo del equipo de EG, además del contenido específico de la ecografía. También se incluye una breve revisión anatómica de la vejiga con sus planos anatómicos para su visualización y la técnica de medición del volumen vesical. Es importante mencionar que el grupo de formación está compuesto por entre 5 y 10 enfermeros, con el objetivo de equilibrar adecuadamente el número de participantes en relación con el número de instructores, así como la disponibilidad del equipo de EG y de voluntarios para la práctica.

Tras la parte teórica, se lleva a cabo una parte práctica estructurada en actividades *prácticas*, con una duración de dos horas, para el desarrollo de habilidades en el manejo del equipo de EG y en la aplicación de la técnica de POCUS vesical en voluntarios. En esta fase, los profesionales son acompañados por una enfermera de referencia en POCUS en la institución, quien también imparte la parte teórica.

Durante el tiempo de formación práctica, se utilizan dos voluntarios de la propia institución, generalmente monitores de la licenciatura en enfermería, a los que se anima a beber agua periódicamente con el fin de mantener la vejiga con volumen de orina para que los enfermeros puedan realizar los exámenes. Cabe destacar que se orienta a los profesionales a realizar entre cinco y diez insonaciones en los voluntarios, acompañados por la instructora mencionada, y después de este momento, se les somete a una evaluación práctica. Esta evaluación consiste en la realización de la técnica de POCUS vesical en uno de los voluntarios, que puede ser considerado apto o no apto por la instructora, basándose en una lista de verificación elaborada por la propia institución que contiene los elementos fundamentales de la técnica correspondiente. La aprobación del profesional en este momento de la formación se produce mediante la suma de aciertos iguales o superiores al 80 % de los elementos presentes en la lista de verificación. Si se considera no apto, el profesional es convocado para una nueva formación con futuras clases y no está autorizado para esta práctica en su unidad de trabajo hasta su aprobación. Es importante destacar que las unidades elegidas para la recolección de datos del presente estudio, en el período seleccionado, contaban con enfermeros con más de seis meses de experiencia en POCUS vesical.

De acuerdo con el protocolo institucional de POCUS vesical, en situaciones en las que el paciente no presenta diuresis espontánea durante más de seis horas o durante más de cuatro horas después de la retirada del CV, el enfermero debe realizar un examen físico para evaluar la presencia de globo vesical palpable, signos de malestar o distensión abdominal, presencia de ganas de orinar o incluso agitación y sudoración. Tras esta evaluación, el enfermero debe realizar el POCUS vesical con el fin de medir el volumen de la vejiga. Dependiendo del volumen encontrado, el protocolo institucional sugiere maniobras de vaciamiento, como ponerse de pie junto a la cama, realizar la maniobra de Valsalva, aplicar calor en la región suprapúbica, hacer sonar agua corriente y sentarse en el inodoro. Si estas medidas no son eficaces o no están indicadas por el cuadro clínico, y el volumen obtenido en el POCUS vesical es superior o igual a 400 ml, o superior a 200 ml acompañado de síntomas de malestar, se llama al médico para discutir la necesidad de una CVA. La CVD está indicada para volúmenes superiores a 1000 ml encontrados en el POCUS vesical y con episodios previos de RU^(18)^. La definición utilizada en este estudio para caracterizar la RU fue un volumen de orina superior a 500 ml encontrado en el POCUS vesical^(2)^.

Los aparatos de ultrasonido utilizados en la institución son equipos SonoSite M-Turbo®, registrados en la Agencia Nacional de Vigilancia Sanitaria con el número 80022060089 y autorizados para uso profesional y para la atención de la población. Estos equipos están configurados para evaluar imágenes utilizando sondas convexas, lineales y sectoriales. Cada una de las unidades mencionadas anteriormente (UTI, semi-intensiva y clínica médico-quirúrgica) dispone de este equipo para uso del personal de enfermería. Para realizar la técnica de medición del volumen vesical utilizada en la institución, el paciente debe colocarse en decúbito dorsal horizontal. A continuación, se coloca el transductor convexo en la región suprapúbica en el plano transversal. En ese momento, al insonar la vejiga, se calcula la longitud del diámetro latero-lateral de la vejiga. Posteriormente, se cambia la posición del transductor, manteniéndolo en la ubicación anatómica, para su visualización en el plano longitudinal. En esta etapa, la insonación de la vejiga debe incluir el cálculo de las longitudes de los diámetros anteroposterior y craneocaudal. El equipo utilizado en la institución calcula automáticamente el volumen vesical.

### Muestra y Criterios de Selección

La muestra fue por conveniencia, compuesta por historias clínicas de pacientes mayores de 18 años, evaluados con POCUS vesical por el enfermero, que no presentaron diuresis espontánea durante más de seis horas o después de la retirada del CV durante más de cuatro horas y a los que se les introdujo el CVA o el CVD después del procedimiento, durante su ingreso en las unidades mencionadas, en el período comprendido entre enero de 2017 y diciembre de 2018. Tras la identificación de los pacientes mediante una base de datos, se encontraron 395 individuos elegibles. Se contactó por teléfono a todos los pacientes y, tras la presentación del estudio, los investigadores enviaron el Término de Consentimiento Libre y Esclarecido (TCLE) a través de la plataforma *Google Forms*. De ellos, 25 rechazaron participar y 99 presentaban registros incompletos en los historiales, lo que dio lugar a la inclusión de 211 pacientes en el estudio. Cabe destacar que se contactó con todos los pacientes elegibles.

### Variables del Estudio

Las variables recopiladas incluyeron información demográfica (sexo y edad), características clínicas (diagnóstico de ingreso, antecedentes clínicos, examen físico, y posibles medicamentos en uso que interfieren en el vaciamiento vesical) y datos relacionados con la realización del POCUS (tiempo de ausencia de micción, motivo de la realización de la EG, volumen de orina encontrado en la EG, conductas tras la realización de la EG y volumen de orina drenado posteriormente por CVA o CVD).

### Recolección de Datos

La recolección de datos fue realizada por los autores mediante la revisión de los historiales electrónicos, tras la autorización de los participantes mediante la firma del TCLE. La identificación de los historiales se realizó en enero de 2020, el contacto con los pacientes se llevó a cabo entre marzo y abril de 2020, y la recolección de datos mediante la revisión de los historiales tuvo lugar entre mayo y junio del mismo año.

### Análisis y Tratamiento de los Datos

Los datos se introdujeron en la plataforma *Research Electronic Data Capture*® (REDCap®)^(19)^ y se importaron a una hoja de cálculo del programa *Microsoft Excel* ® 2007. Para los análisis se utilizó el software *Statistical Package for the Social Science* (SPSS), versión 26. El análisis descriptivo se realizó mediante frecuencias absolutas y relativas para las variables cualitativas, mientras que para las cuantitativas se comprobó primero la normalidad mediante la prueba de *Shapiro-Wilk.* Tras verificar la normalidad, se calcularon la media y la desviación estándar. En cuanto al análisis inferencial, se utilizaron las pruebas de chi-cuadrado y *Fisher* para las variables cualitativas y la prueba T de *Student* para las cuantitativas.

Para analizar la comparación entre el volumen vesical observado en la POCUS por el enfermero y el volumen de orina encontrado posteriormente en la CVA o CVD, se utilizó el coeficiente de correlación de *Pearson*. El nivel de significación adoptado para el análisis fue de *p* ≤ 0,005.

### Aspectos Éticos

La presente investigación se realizó de acuerdo con la resolución n.º 466/2012, y fue sometida y aprobada por el Comité de Ética en Investigación de dicha institución, con el número de aprobación 4.533.418 y CAEE: 40058020700000071.

## RESULTADOS

Se incluyeron un total de 211 pacientes, a los que se les realizó 211 POCUS vesicales por parte del enfermero (uno por paciente). En la Tabla 1 se muestra la caracterización de los pacientes sometidos a la realización del POCUS vesical por parte del enfermero según la presencia o ausencia de RU. La RU estuvo presente en 119 (56,3 %) de los pacientes, de los cuales más de la mitad eran varones, con una media de edad de 65,1 ± 20,2 años. La hipertensión arterial sistémica (HAS) fue el antecedente clínico más común, y el diagnóstico de ingreso hospitalario postoperatorio inmediato fue más frecuente en el grupo con RU. Entre los medicamentos en uso con potencial para alterar la frecuencia urinaria, destacan los opioides en pacientes con RU.

**Tabla 1:** Caracterización de los pacientes que fueron sometidos a la realización de POCUS vesical por el enfermero según la presencia o ausencia de RU – São Paulo, SP, Brasil, 2020.

Variables	Total n = 211 (%)	Sin RU n = 92 (%)	Con RU n = 119 (%)	Valor de *p*
Sexo				
Masculino	123 (58,3)	51 (55,4)	72 (60,5)	0,459^a^
Femenino	88 (41,7)	41 (44,6)	47 (39,5)	
Antecedentes clínicos				
HAS	81 (38,4)	41 (44,6)	40 (33,6)	0,105^a^
DM	30 (14,2)	14 (15,2)	16 (13,4)	0,715^a^
EPOC	14 (6,6)	9 (9,8)	5 (4,2)	0,106^a^
IC	14 (6,6)	9 (9,8)	5 (4,2)	0,106^a^
ERC	8 (3,8)	4 (4,3)	4 (3,4)	0,710^a^
Diagnóstico de internación				
Post-operatorio	103 (48,8)	35 (38,0)	68 (57,1)	0,006^a^
Neurológico	42 (19,9)	24 (26,1)	18 (15,1)	0,048^a^
Respiratorio	25 (11,8)	15 (16,3)	10 (8,4)	0,078^a^
Infeccioso	22 (10,4)	9 (9,8)	13 (10,9)	0,788^a^
Cardiovascular	13 (6,2)	4 (4,3)	9 (7,6)	0,335^a^
Urológico	12 (5,7)	7 (7,6)	5 (4,2)	0,289^a^
Gastrointestinal	7 (3,3)	3 (3,3)	4 (3,3)	0,748^a^
Clase farmacológica				
Opioide	63 (29,9)	18 (19,6)	45 (37,8)	0,004^a^
Antipsicótico	38 (18,0)	18 (19,6)	20 (16,8)	0,605^a^
Analgésico narcótico	36 (17,1)	16 (17,4)	20 (16,8)	0,911^a^
Antiespasmódico	27 (12,8)	14 (15,2)	13 (10,9)	0,355^a^
Antidepresivo	14 (6,6)	6 (6,5)	8 (6,7)	0,954^a^
Antiparkinsoniano	11 (5,2)	5 (5,4)	6 (5,0)	> 0,999^b^
Óbito	49 (23,2)	26 (28,3)	23 (19,3)	0,128^a^

RU: retención urinaria, HAS: hipertensión arterial sistémica; DM: diabetes mellitus; EPOC: enfermedad pulmonar obstructiva crónica; IC: insuficiencia cardíaca; ERC: enfermedad renal crónica; UTI: unidad de tratamiento intensivo, a: prueba de chi cuadrado, b: prueba exacta de *Fisher*.

La información relacionada con la realización de la ecografía vesical y la micción según la presencia o ausencia de RU se recoge en la Tabla 2. La unidad que más ecografías vesicales realizó fue la unidad de medicina y cirugía. La presencia de globo vesical palpable fue más frecuente en el grupo con RU, y el motivo predominante para la ausencia de micción que llevó a la realización del POCUS por parte del enfermero estuvo asociado con la realización de procedimientos quirúrgicos. El volumen vesical encontrado en el POCUS fue significativamente superior en los pacientes con RU. La principal acción tras la realización del POCUS vesical fue la introducción del CV, y el volumen de orina drenado por el CVA o CVD tras el POCUS fue mayor en el grupo con RU.

**Tabla 2:** Información relacionada con la realización del POCUS vesical y la micción según la presencia o ausencia de RU – São Paulo, SP, Brasil, 2020.

Variable	Total (n = 211)	Sin RU (n = 92)	Con RU (n = 119)	Valor de *p*
Unidad de realización do POCUS				
Clínica médico-quirúrgica	142 (67,3)	56 (60,9)	86 (72,3)	0,080^a^
Semi-intensiva	36 (17,1)	19 (20,7)	17 (14,3)	0,223^a^
UTI	33 (15,6)	17 (18,5)	16 (13,4)	0,318^a^
Tiempo sin orinar (horas)				
Media ± DE	7,5 ± 2,5	7,6 ± 2,4	7,4 ± 2,5	0,586^b^
Globo vesical palpable n (%)	113 (56,3)	38 (41,3)	75 (63,0)	0,002^a^
Causas da ausencia de micción				
Procedimiento quirúrgico n (%)	75 (35,5)	23 (25,0)	52 (43,7)	0,005^a^
Retirada de CVD n (%)	74 (35,1)	40 (43,5)	34 (28,6)	0,024^a^
RNC n (%)	29 (13,7)	19 (20,7)	10 (8,4)	0,010^a^
Obstrucción de CVD n (%)	2 (0,9)	1 (1,1)	1 (0,8)	> 0,999^c^
Volumen verificado en el POCUS				
Media ± DE	563,3 ± 251,8	345,0 ± 107,5	732,1 ± 195,0	< 0,001^b^
Acción después del POCUS				
CVA n (%)	190 (90,0)	81 (88,0)	109 (91,5)	0,544^a^
CVD n (%)	21 (10,0)	10 (10,9)	11 (9,2)	0,696^a^
Volumen drenado por CVA o CVD después de POCUS				
Media ± DE	593,1 ± 290,3	395,0 ± 167,3	746,3 ± 272,4	< 0,001^b^

RU: retención urinaria, POCUS: *point of care ultrasonography* (ecografía en el punto de atención), UTI: unidad de tratamiento intensivo, CVD: catéter vesical de demora, RNC: disminución del nivel de conciencia, CVA: catéter vesical de alivio, a: prueba de chi cuadrado, b: prueba T de *Student*, c: prueba exacta de *Fisher*.

En la Figura 1, se puede observar la correlación entre los volúmenes estimados por el POCUS realizado por el enfermero y el volumen de orina verificado posteriormente mediante la introducción del CVA o CVD. En la Figura 1A, se observa la correlación entre el volumen del POCUS hasta 200 ml y el volumen por CVA o CVD (r = 0,759, *p* < 0,001). La Figura 1B muestra la correlación entre el volumen del POCUS de 200 a 500 ml y el volumen por CVA o CVD (r = 0,836, *p* < 0,001). La Figura 1C muestra la correlación entre el volumen del POCUS por encima de 500 ml y el volumen por CVA o CVD (r = 0,922, *p* < 0,001). Por último, la Figura 1D ilustra la correlación entre el volumen del POCUS, sin tomar en cuenta la cantidad de ml encontrados, y el volumen por CVA o CVD (r = 0,958, *p* < 0,001).

**Figura 1:** Correlación entre los volúmenes del POCUS y el volumen de orina verificado en la posterior introducción del CVA o CVD – São Paulo, SP, Brasil, 2020.

POCUS: *point of care ultrassonography*, CVA cateter vesical de alivio, CVD: cateter vesical de demora, ml: mililitros.

La correlación entre los volúmenes estimados en el POCUS vesical realizado por el enfermero, según la unidad de hospitalización, y el volumen de orina verificado posteriormente mediante la introducción del CVA o CVD se muestra en la Figura 2. En la Figura 2A se observa la correlación entre el volumen del POCUS vesical encontrado por los enfermeros de la clínica médico-quirúrgica y el volumen de orina drenado por CVA o CVD (r = 0,959, *p* < 0,001). La Figura 2B muestra la correlación entre el volumen del POCUS vesical medido por los enfermeros de cuidados semiintensivos y el volumen drenado por CVA o CVD (r = 0,985, *p* < 0,001). La Figura 2C muestra la correlación entre el volumen del POCUS vesical insonado por enfermeros de la UTI y el volumen drenado por CVA o CVD (r = 0,940, *p* < 0,001).

**Figura 2:** Correlación entre los volúmenes de POCUS por parte del personal de enfermería, según la unidad, y el volumen de orina verificado tras la posterior introducción del CVA o CVD. En la Figura 1A se muestra la correlación entre el volumen de POCUS vesical encontrado por los enfermeros de la clínica médico-quirúrgica, en la Figura 1B por los enfermeros de la semi-intensiva y en la Figura 1C por los enfermeros de la UTI – São Paulo, SP, Brasil, 2020.

POCUS: *point of care ultrasonography*, CVA cateter vesical de alívio, CVD: cateter vesical de demora, ml: mililitros.

## DISCUSIÓN

En el presente estudio se puede observar un perfil de pacientes de edad avanzada, la mayoría de los cuales se sometieron a algún tipo de procedimiento quirúrgico que requirió la evaluación del enfermero por no presentar diuresis, principalmente tras la retirada del CVD. La correlación entre el volumen encontrado en el POCUS vesical por el enfermero y el volumen de orina drenado por CVA o CVD, en general, fue significativamente fuerte, ya que se modifica gradualmente según la cantidad de volumen encontrada en el POCUS, y no presenta diferencias según el lugar de trabajo del enfermero.

En relación con la prevalencia de RU, más de la mitad de los pacientes analizados presentaron esta alteración. En una revisión de alcance brasileño, se mostró que, entre las principales prácticas utilizadas para el diagnóstico de RU, destaca el uso de EG para la toma de decisiones frente al CV^(20)^. En un estudio holandés con pacientes en postoperatorio de cirugía de artroplastia de cadera y rodilla, la frecuencia de RU fue significativamente inferior a la del presente estudio^(21)^. Sin embargo, en un estudio polaco con pacientes en el mismo contexto quirúrgico, la prevalencia encontrada fue similar a la de nuestra investigación^(22)^. En el escenario nacional, un estudio con pacientes de una clínica médico-quirúrgica presentó una tasa de RU inferior^(1)^, sin embargo, en otro estudio brasileño reciente, con pacientes críticos, la tasa de RU encontrada fue similar a la de la presente investigación^(2)^. La variedad entre las prevalencias de RU mencionadas puede atribuirse a diferencias en los protocolos asistenciales, en las características clínicas de las poblaciones estudiadas, como la edad, las comorbilidades asociadas (como hipertensión y diabetes), el estado crítico de salud, el tipo de cirugía realizada y el tipo de anestesia aplicada, as bien que en los criterios diagnósticos utilizados y en la aplicación de intervenciones preventivas, como el uso de ecografía vesical y estrategias de manejo individualizado^(1,2,21,22)^.

La ausencia de micción durante un determinado período está relacionada con una posible RU y determina la decisión del enfermero de realizar la insonación. En este sentido, el conocimiento previo de los motivos que provocan una reducción de la actividad miccional es esencial para la realización precoz del POCUS vesical y para proporcionar beneficios al paciente^(23)^. En el presente estudio se observa que el período postoperatorio es un momento preponderante para la reducción de la micción y el desarrollo de la RU. Entre los factores intrínsecos relacionados con este período se encuentran los tipos de procedimientos quirúrgicos, con especial destaque a las cirugías ortopédicas, que presentan una frecuencia significativa de incidencia de RU^(20)^, además de que la duración de la cirugía superior a cuatro horas llega a duplicar el riesgo de RU^(21)^. En un estudio estadounidense con pacientes sometidos a artroplastia total de rodilla, se demostró que determinados fármacos, en este caso antiinflamatorios no esteroideos y glicopirrolato, administrados en el período intraoperatorio son predictores del riesgo de desarrollo de RU, con un aumento del riesgo de RU de hasta cuatro veces^(24)^.

Otro factor relevante relacionado con la ausencia de micción espontánea es el periodo posterior a la retirada del CVD. Un ensayo clínico estadounidense comparó el impacto de la creación de un protocolo para la retirada precoz del CVD en pacientes postoperatorios. Se observó un aumento de la RU (12,4 % a 26,7 %, *p* = 0,003), sin embargo, hubo una reducción en la tasa de ITU (3,0 % a 1,0 %, *p* = 0,280)^(25)^. En una revisión sistemática y metaanálisis de 2020, que comparó la retirada precoz del CVD con la tardía, en relación con la RU y la ITU, se estableció que los estudios son inconclusos para comparar la inferioridad de la retirada precoz frente a la tardía del CVD en relación con la RU. Sin embargo, la retirada precoz es superior en términos de reducción del riesgo de ITU^(26)^.

Es fundamental crear protocolos para reducir el CV innecesaria mediante el uso de POCUS. En un estudio estadounidense sobre el protocolo de cateterización innecesaria en el postoperatorio, que incluye la retirada del DVC hasta seis horas después de la operación, con la participación del enfermero a través del POCUS para medir el contenido urinario y limitar el CV solo al volumen encontrado en el EG por encima de 150 ml, demostró una reducción del 90,0 % de las cateterizaciones innecesarias, además de acelerar el flujo de altas hospitalarias (4,0 % a 22,0 %, *p* = 0,022)^(27)^.

A pesar de la escasez de evidencia en la literatura sobre la precisión y la capacitación del enfermero en el uso del POCUS vesical, un estudio con cinco tipos de simuladores que representaban diferentes situaciones clínicas demostró una excelente correlación entre los volúmenes medidos por el enfermero y los volúmenes reales, aunque no hubo estratificación por gradaciones de volumen o por situación clínica^(28)^.

En este sentido, un estudio estadounidense con enfermeros recién habilitados en POCUS vesical evaluó la correlación entre los volúmenes estimados por EG y el volumen urinario drenado por CV, e identificó una fuerte correlación en las primeras insonaciones, con una correlación muy fuerte a partir de la segunda y tercera medición^(29)^. El único estudio brasileño que abordó este tema evidenció una correlación muy fuerte entre los volúmenes, sin embargo, no hubo estratificación por la cantidad de volúmenes o por la unidad de trabajo del enfermero^(1)^.

En un estudio francés, también se encontró una correlación muy fuerte entre los volúmenes analizados y una excelente precisión según la curva característica de funcionamiento del receptor (Receiver Operating Characteristics ROC) para volúmenes superiores a 600 ml, lo que corrobora los hallazgos del presente estudio en relación con la excelente evaluación de la ecografía vesical por parte del enfermero en volúmenes superiores a 500 ml^(30)^. Cabe mencionar que la literatura es limitada en cuanto a los factores intrínsecos a la práctica de la insonar que dificultan la realización de la POCUS vesical por parte del enfermero.

Las implicaciones de los resultados de este estudio para la práctica de la enfermería son sustanciales. El uso de la ecografía vesical por parte de los enfermeros puede optimizar la atención a los pacientes, permitiendo intervenciones rápidas y precisas que mejoran significativamente los resultados clínicos. La capacidad de identificar y cuantificar la RU con un método no invasivo y eficaz puede reducir la incidencia de complicaciones asociadas, como la ITU y la prolongación de la hospitalización. Por lo tanto, es fundamental que los protocolos de asistencia de enfermería implementen el uso del POCUS vesical en diferentes escenarios y contextos.

En el ámbito de la enseñanza, es imperativo que la formación de los enfermeros incluya módulos específicos sobre el uso del POCUS. La implementación de cursos teóricos y prácticos estructurados durante la licenciatura y en programas de educación continua puede capacitar a los profesionales de la salud para utilizar esta herramienta de manera eficaz y segura. Además, se debe fomentar el desarrollo de habilidades en POCUS mediante simuladores y escenarios clínicos que reproduzcan situaciones reales, garantizando que los enfermeros estén preparados para aplicar estos conocimientos en la práctica clínica.

Para los efectos de investigación, este estudio abre diversas oportunidades para futuras investigaciones que podrían explorar la relación entre el uso del POCUS y otros resultados clínicos, además de investigar el impacto económico de la implementación de esta tecnología en la práctica de enfermería. Sin embargo, la investigación actual presenta algunas limitaciones al tratarse de un estudio retrospectivo, lo que puede introducir sesgos en la recolección e interpretación de los datos. Además, la muestra estaba compuesta por pacientes de un único centro hospitalario, lo que puede limitar la generalización de los resultados.

## CONCLUSIÓN

Los resultados demostraron que las principales indicaciones para la realización del POCUS fueron la ausencia de micción después de procedimientos quirúrgicos, la retirada del CVD, la presencia de RNC y la sospecha de obstrucción del CVD. El volumen encontrado por el POCUS vesical realizado por el enfermero presentó una correlación muy fuerte con el volumen de orina drenado por CVA o CVD, y que además esta correlación aumenta gradualmente según el volumen. Además, la correlación no varió entre las diferentes unidades de trabajo de los enfermeros. Estos hallazgos refuerzan la importancia de incluir el POCUS en los protocolos de asistencia de enfermería, con el fin de promover una asistencia más cualificada y segura a los pacientes.
